# Concurrent Endometrial Cancer in Women with Atypical Endometrial Hyperplasia: What Is the Predictive Value of Patient Characteristics? †

**DOI:** 10.3390/cancers16010172

**Published:** 2023-12-29

**Authors:** Luca Giannella, Francesco Piva, Giovanni Delli Carpini, Jacopo Di Giuseppe, Camilla Grelloni, Matteo Giulietti, Francesco Sopracordevole, Giorgio Giorda, Anna Del Fabro, Nicolò Clemente, Barbara Gardella, Giorgio Bogani, Orsola Brasile, Ruby Martinello, Marta Caretto, Alessandro Ghelardi, Gianluca Albanesi, Guido Stevenazzi, Paolo Venturini, Maria Papiccio, Marco Cannì, Maggiorino Barbero, Massimiliano Fambrini, Veronica Maggi, Stefano Uccella, Arsenio Spinillo, Francesco Raspagliesi, Pantaleo Greco, Tommaso Simoncini, Felice Petraglia, Andrea Ciavattini

**Affiliations:** 1 Woman’s Health Sciences Department, Gynecologic Section, Polytechnic University of Marche, 60123 Ancona, Italy; lucazeta1976@libero.it (L.G.); giovanni.dellicarpini@ospedaliriuniti.marche.it (G.D.C.); jacopo.digiuseppe@ospedaliriuniti.marche.it (J.D.G.); c.grelloni@pm.univpm.it (C.G.); 2 Department of Specialistic Clinical and Odontostomatological Sciences, Polytechnic University of Marche, 60131 Ancona, Italy; f.piva@staff.univpm.it (F.P.);; 3 Gynecologic Oncology Unit, IRCCS—Centro di Riferimento Oncologico di Aviano, 33081 Aviano, Italy; fsopracordevole@cro.it (F.S.); ggiorda@cro.it (G.G.); anna.delfabro@cro.it (A.D.F.); nicolo.clemente@cro.it (N.C.); 4 Department of Obstetrics and Gynecology, Fondazione IRCCS Policlinico San Matteo, Università degli Studi di Pavia, 27100 Pavia, Italy; barbara.gardella@unipv.it (B.G.); spinillo@smatteo.pv.it (A.S.); 5 Gynecological Oncology Unit, Fondazione IRCCS—Istituto Nazionale Tumori, 20133 Milano, Italy; giorgio.bogani@istitutotumori.mi.it (G.B.); raspagliesi@istitutotumori.mi.it (F.R.); 6 Section of Obstetrics and Gynecology, Department of Medical Sciences, University of Ferrara, 44124 Ferrara, Italy; brsrsl@unife.it (O.B.); mrtrby@unife.it (R.M.); grcptl@unife.it (P.G.); 7 Division of Obstetrics and Gynecology, Department of Clinical and Experimental Medicine, University of Pisa, 56124 Pisa, Italy; marta.caretto@phd.unipi.it (M.C.); tommaso.simoncini@med.unipi.it (T.S.); 8 UOC Ostetricia e Ginecologia, Ospedale Apuane, Azienda Usl Toscana Nord-Ovest, 54100 Massa, Italy; alessandro.ghelardi@uslnordovest.toscana.it (A.G.);; 9 Department of Obstetrics and Gynaecology, ASST Ovest MI, Legnano (Milan) Hospital, 20025 Legnano, Italy; guido.stevenazzi@asst-ovestmi.it; 10 Division of Obstetrics and Gynecology, AUSL di Modena, 41012 Carpi, Italy; p.venturini@ausl.mo.it (P.V.); m.papiccio@ausl.mo.it (M.P.); 11 Department of Obstetrics and Gynecology, Asti Community Hospital, 14100 Asti, Italy; mcanni@asl.at.it (M.C.); barberom@tin.it (M.B.); 12 Obstetrics and Gynecology, Department of Experimental, Clinical, and Biomedical Sciences, Careggi University Hospital, University of Florence, 50121 Florence, Italy; massimiliano.fambrini@unifi.it (M.F.); felice.petraglia@unifi.it (F.P.); 13 Department of Obstetrics and Gynecology, University of Verona, 37129 Verona, Italy; veronica.maggi@studenti.univr.it (V.M.); stefano.uccella@univr.it (S.U.)

**Keywords:** atypical endometrial hyperplasia, endometrial cancer, artificial intelligence, regression models, prediction model, patient characteristics

## Abstract

**Simple Summary:**

The rate of concurrent endometrial cancer (EC) in women with atypical endometrial hyperplasia (AEH) is not negligible. Furthermore, among women with EC, about 12% may have a high-risk disease requiring lymph node status assessment. Given that endometrial sampling cannot exclude EC in women with AEH, knowing variables that increase the risk of malignancy can be helpful in clinical practice. Some patient characteristics were associated with this occurrence, representing possible risk factors on which to adjust treatment planning. No prediction models with internal validation showed the impact of patient characteristics in predicting EC after a preoperative diagnosis of AEH. The present study, using regressions and artificial neural networks, found recurrent patient characteristics in women with EC. However, they likely do not contain good/optimal discriminating information. Future predictive models should include other individual factors (e.g., genotypic variables) to move toward more personalized medicine.

**Abstract:**

Background: The rate of concurrent endometrial cancer (EC) in atypical endometrial hyperplasia (AEH) can be as high as 40%. Some patient characteristics showed associations with this occurrence. However, their real predictive power with related validation has yet to be discovered. The present study aimed to assess the performance of various models based on patient characteristics in predicting EC in women with AEH. Methods: This is a retrospective multi-institutional study including women with AEH undergoing definitive surgery. The women were divided according to the final histology (EC vs. no-EC). The available cases were divided into a training and validation set. Using k-fold cross-validation, we built many predictive models, including regressions and artificial neural networks (ANN). Results: A total of 193/629 women (30.7%) showed EC at hysterectomy. A total of 26/193 (13.4%) women showed high-risk EC. Regression and ANN models showed a prediction performance with a mean area under the curve of 0.65 and 0.75 on the validation set, respectively. Among the best prediction models, the most recurrent patient characteristics were age, body mass index, Lynch syndrome, diabetes, and previous breast cancer. None of these independent variables showed associations with high-risk diseases in women with EC. Conclusions: Patient characteristics did not show satisfactory performance in predicting EC in AEH. Risk stratification in AEH based mainly on patient characteristics may be clinically unsuitable.

## 1. Introduction

Atypical endometrial hyperplasia (AEH) is a premalignant lesion that may reveal concurrent cancer on definitive histology in 40% of cases [1,2]. Moreover, 10–15% of women with endometrial cancer (EC) may have a high-risk disease requiring lymph node status assessment [3,4,5]. Current guidelines suggest nodal staging should be performed in intermediate-high-risk/high-risk diseases. The sentinel lymph node technique represents an acceptable alternative to systematic lymphadenectomy for lymph node staging in stages 1 and 2 [6].

It is known that these women often do not undergo oncological work-up, and definitive histology may reveal a stage of the disease that would have required more radical surgery [7]. Moreover, it should be considered that conservative treatment may be an option in women of childbearing age [6]. Therefore, an accurate diagnosis is of pivotal importance.

Some EC predictors may affect the rate of unexpected cancer in AEH [8,9,10,11,12]. Matsuo et al. studied 211 women with EH and showed that older age, obesity, diabetes mellitus, and complex atypical hyperplasia were associated with concurrent EC in AEH [10]. Given that endometrial sampling cannot exclude EC in women with AEH, knowing those variables that increase the risk of malignancy can be helpful in clinical practice.

Although some independent variables showed associations with cancer, there have been no attempts to build prediction models with related validation. In the latter studies, multivariate analyses were used to assess only the presence or absence of associations between independent variables and the presence of EC in women with a previous diagnosis of AEH [8,9,10,11,12]. Testing independent variables in the training and validation set would allow us to test the real predictive power of patient characteristics to assess their clinical utility. Moreover, the study of variables in the sub-population of women with high-risk diseases would enable us to measure their impact in identifying patients needing more radical surgery.

The present study aimed to create and assess prediction models of EC in women with AEH using various regression and artificial intelligence (AI) methods.

## 2. Materials and Methods

### 2.1. Study Design and Setting

This retrospective observational study included women with a preoperative diagnosis of AEH and then undergoing definitive surgery within 40 days between January 2015 and December 2020. The histological reference standard was represented by hysterectomy. Women with incomplete data, previous events of AEH managed with medical treatment, and AEH diagnosis on endometrial structural lesions (e.g., polyps) were excluded.

Based on Italian law, the Ethics Committee (Comitato Etico Regionale Marche) took note of the study (Prot. 338/2021) [13]. According to Italian law, patient consent was not mandatory in a retrospective study [13].

### 2.2. Variables

The histological classification of AEH refers to the WHO 2014 Classification (atypical vs. non-atypical) [14,15].

Patient characteristics taken into account were age, parity, menopausal status, body mass index (BMI), presence of hypertension, or diabetes, Lynch syndrome, hormonal therapy, smoking, previous tamoxifen therapy (previous use of Tamoxifen for 5 years), abnormal uterine bleeding, previous breast cancer, and endometrial sampling method (D&C: dilation and curettage, HSC-bio: hysteroscopically guided biopsy, and HSC-res: hysteroscopically endometrial resection).

Descriptive patient characteristics also included the type of surgery (laparotomic, laparoscopic, vaginal). According to the classification system for EC, women were classified as low-risk, intermediate-risk, high-intermediate-risk, and high-risk [6].

### 2.3. Sample Size Calculation

Based on previous data for prediction models, we considered a minimum of 10 events (EC) for each predictor variable [16]. Since we included 16 independent variables, the minimum required sample size should consist of 160 cases of EC.

### 2.4. Statistical Methods

The final histology (dependent variable) divided the women into patients with or without cancer. The Kolmogorov–Smirnov test was used to test continuous variable distribution. The univariate analysis was used to compare all studied independent variables. The non-parametric Mann–Whitney U-test for two independent samples was used for not normally distributed values. Comparisons between categorical variables were performed using the Chi-squared test.

The performance of endometrial cancer prediction methods was assessed according to Table S1 scheme. The cancer prediction models were trained and tested on the definitive histological reference standard (hysterectomy). All statistical analyses were performed using MATLAB Software (MATLAB R2022a, The MathWorks, Inc., Natick, MA, USA). *p* < 0.05 was considered to indicate statistical significance.

Apart from the age and BMI variables, all the others were considered categorical. They were assigned the value 0 (absent) or 1 (present) and passed through the “categorical” function of Matlab. The regression models were implemented using the Matlab functions “fitlm”, “fitglm”, and “mnrfit”. We specify that Matlab’s “fitlm” function can handle categorical variables when the options “bisquare” or “logistic” or “fitglm” function was used with the “Distribution: binomial” option.

Regarding regressions, we also used the parameter “linear” for linear regression (“model contains an intercept and linear term for each predictor”), or “interactions” that is a model containing an intercept, a linear term for each predictor, and all products of pairs of distinct predictors (no squared terms) or “pure quadratic” that is a model containing an intercept term and linear and squared terms for each predictor, or “quadratic” for quadratic regression with interactions. We used 10-fold cross-validation in which the train set contained 80% of the cases and the test set 20%. This was implemented using the “cvpartition” function with the “kfold” option. In particular, we wanted to assess which variables had the most discriminating power to reduce the dimensionality of the model.

Other predictors were constructed using artificial neural network (ANN) algorithms using the “patternnet” Matlab function. After running several tests with different nodes, we built predictive models with many nodes proportional to the number of variables in the model. In this way, the complexity was proportional to the number of variables. We found the configurations that provided the best performance. Also, for ANN, we have implemented 10-fold cross-validation. We also used the SVM (support vector machine) and RF (random forest) AI algorithms.

Regarding the prediction models obtained on the same variables, we showed the five combinations of variables associated with the best AUC. Finally, recurrent patient characteristics were considered those variables present in at least four of five prediction models.

## 3. Results

### 3.1. Study Population

**Figure 1:**
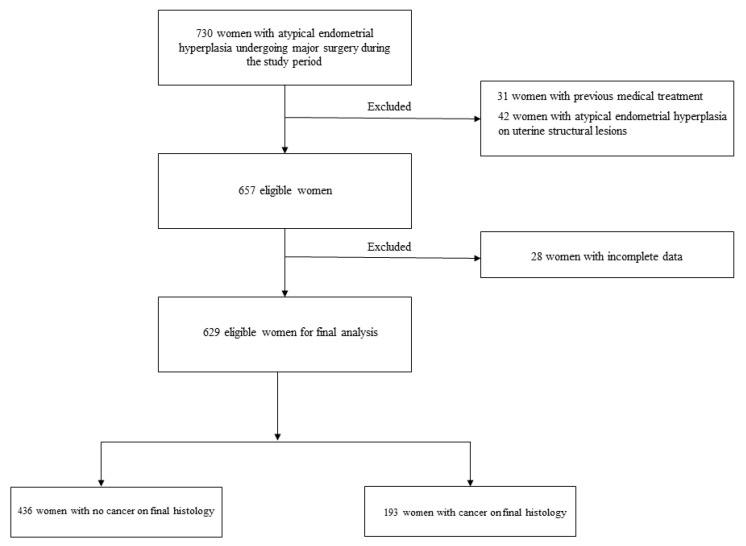
Study flow-chart.

The study period included 730 consecutive women with AEH undergoing major surgery. After excluding 101 cases, 629 eligible women were analyzed (Figure 1). Patient characteristics are reported in Table 1. The surgical approach is reported in Table S2. Univariate analysis results comparing women with or without cancer on hysterectomy are reported in Table 2.

From here on, we built models to predict whether AEH patients also had concomitant EC. Although Table 2 showed variables not significantly related to cancer, we initially decided not to discard any variables since the models can capture correlations between unrelated variables to cancer.

### 3.2. Regression-Based Predictors

To test the real predictive power of different kinds of regressions (linear, interactions, quadratic, and purequadratic), we trained the same models across multiple data sets (training) and tested them on different sets (test). In particular, we wanted to assess which variables had the most discriminating power to reduce the dimensionality of the model. We then performed the linear regression with all numbers and combinations of variables. Regarding the regressions performed on the same variables, we showed the five combinations of variables associated with the best AUC (Table S3). It is noted that the AUC calculated in the train set increased as the number of variables increased. This is perfectly normal and does not necessarily imply a good performance in prediction. The AUC calculated on the test sets increased until it reaches the maximum of about 0.65 in correspondence with a 5-variable model, among which the most common were age, BMI, Lynch syndrome, abnormal uterine bleeding, and previous breast cancer. Linear regression models that used more than these five variables did not improve performance or even progressively deteriorated it despite increasing the AUC of the training set.

We also tested whether a quadratic regression improved performance thanks to capturing nonlinear interactions between variables (Table 3). This model achieved the maximum AUC (0.65) in the test set using a combination of four variables. The most recurrent patient characteristics were age, BMI, and Lynch syndrome. However, the best linear and quadratic regressions yielded a similar performance with a sensitivity and a specificity of around 61%.

We did not report the results of the interactions and pure quadratic regressions because they returned slightly lower performance than the previous tests.

### 3.3. Artificial Intelligence-Based Predictors

To assess if better performance could be obtained, we have adopted algorithms (“patternnet” function) that use artificial intelligence to capture deeper non-linearities present in the data, providing greater efficiency than regression algorithms. We tested networks with a proportional number of nodes for each number of variables N (from 2 to 15). For example, from 2*N to 5*N nodes. The best results were obtained with 3*N nodes (Table 4) reaching an AUC in the test set of about 0.75, including ten variables. Sensitivity ranged from 66% to 76%, while specificity ranged from 59% to 67%. These values may not be considered satisfactory for a prediction model. The most recurrent patient characteristics among the best five predictive models were: age, body mass index, Lynch syndrome, diabetes, and previous breast cancer. The performance did not improve significantly with a higher number of nodes. Unfortunately, with a slight improvement compared to the regression models, these use many more variables (i.e., 10 to 12). In this last case, the AUC reached a value of 0.76 (CI 95%: 0.706–0.814), with a sensitivity and specificity of 68.1% and 68.9%, respectively. These results demonstrate a relationship between the indicated variables and the EC, but they do not reach significant values for a clinical impact. We also tested a feedforward, fully connected neural network for regression implemented using the “fitrnet” Matlab function, but the performance was slightly lower than the previous algorithm (patternnet).

Support Vector Machine (SVM)-based prediction algorithms showed disappointing performance, as seen from the AUC (Table S4). Finally, Random Forest (RF) based models performed worse than SVMs.

It is to underline that the results of the three regression functions “fitlm”, “fitglm”, “mnrfit” were almost identical, as shown in the comparative Figure S1.

### 3.4. Recurrent Patient Characteristics in Women with High-Risk Disease

EC’s most recurrent patient characteristics were compared between women with high-risk versus no high-risk disease. There were no statistically significant differences in their distribution between the two groups (Table 5).

## 4. Discussion

The current study, including an extensive sample of women, showed that the performance of patient characteristics in predicting EC in patients with AEH using regression and ANN models could have been better. The best AUC in the test set was about 0.75, which cannot be considered enough for a clinical impact model. Generally, in clinical practice, an AUC of at least 0.8 is considered acceptable [17,18,19]. Finally, the most recurrent variables showed no associations with high-risk diseases in women with EC.

The assessment of various predictive models for EC in women with AEH and their validation using more straightforward and complex algorithms is new. Therefore, we cannot rely on other predictive models to evaluate whether the present performance is better or worse. Overall, our models could be more satisfactory.

The percentage of concurrent cancer found at final histology is similar to previous studies [8,9,10,11,12]. Most women with cancer had low-risk EC. An unrecognized low-risk EC would not lead to significant differences in treatment compared to AEH. However, AEH includes total hysterectomy as a standard surgical treatment, while EC provides total hysterectomy with salpingo-oophorectomy [6]. Ovarian preservation is only offered in women < 45 years of age with low-grade EC in the absence of hereditary cancer syndromes [6]. Likewise, if the conservative hormonal option is chosen, women should know that the recurrence rates are 40.6% in EC G1 vs. 26% in AEH [20]. Our results also showed that the rate of high-risk diseases worthy of lymph node assessment was 13%. Previous studies showed a 12% rate of high-grade EC and deep myometrial invasion [9]. Based on the above, the importance of defining the correct diagnosis in this area is evident. Risk stratification needs to know when to refer these patients to a gynecological oncologist to manage better women deserving of more radical surgery and not delay a possible adjuvant treatment.

Previous authors have posed the question by evaluating clinical variables associated with this occurrence [8,9,10,11,12]. Some patient characteristics, such as BMI, older age, and diabetes, were associated with EC at final histology [8,9,10,11,12]. According to these results, several authors suggested that in the presence of these risk factors, one should think about the existence of cancer and modify the management of AEH accordingly [8,9,10,11,12]. However, our results showed that their predictive power was clinically unsuitable in predicting EC when tested on the validation set. An earlier correspondence already showed how a strong association between BMI > 30 and EC ultimately provided small changes in the post-test probability compared to the pre-test probability, ranging from 4.9% to 7.4% [21,22].

It is difficult to explain why a woman with AEH then has an EC at hysterectomy, especially if she undergoes surgery within a short period (40 days). The woman may be in an advanced stage of the AEH transformation process to explain the evolution into an invasive form shortly after that. The most apparent reason may be an endometrial sampling error. Our data showed that endoscopic endometrial sampling had a lower underestimation of EC than D&C (28% vs. 35%, respectively). In this regard, in women with AEH, endometrial sampling procedures should prefer a hysteroscopic assessment, especially in patients who are candidates for fertility-sparing treatment [6].

The explanation of our results cannot be separated from further research implications. It is known that there are estrogen-related forms of EC in which BMI and other clinical variables play a pivotal role [23]. On the other hand, there are non-estrogen-dependent ECs where the patient characteristics are less decisive [23]. The Proactive Molecular Risk Classifier for Endometrial Cancer (ProMisE) groups stratified different phenotypes [24]. The POLE-mt women were younger and had the lowest BMI, most frequently at FIGO stage 1. The p53-wt group includes women with high BMI. The p53-abn women have advanced age, with a high prevalence of adjuvant treatment and less frequently at FIGO stage 1. Finally, MMR-d women provided intermediate values for all clinical features between the four ProMise groups [24]. This clinical characterization can suggest different pathogenetic mechanisms and explain how the same patient characteristics at different values may identify different sub-populations of women with EC. Likewise, this may explain why such independent variables may appear to have little discrimination in prediction models when genetic mutation assessment is not included.

Recent studies added information on the progression model of endometrial hyperplasia to cancer [23]. It is well known that the evolution of cancer is driven by estrogenic stimulation. However, new evidence suggests a crucial role of some gene mutations (PTEN, PIK3CA, FGFR2, ARID1A, and MYC) in cancer progression from endometrial hyperplasia [23]. In endometrial hyperplasia progressing to cancer, more mutations in oncogenic signaling pathways have been found as occurs in endometrial cancer [23]. We know that areas of AEH may coexist in women with EC. Also, for AEH, a gene panel assessment could be added to standard histological evaluation to better discriminate those cases of unrecognized ECs and modify the management accordingly.

Blood chemical markers could represent further predictive factors to be investigated. Increased preoperative and postoperative D-Dimer levels predicted high-risk EC [25]. Furthermore, the combination of HE4, d-dimer, fibrinogen, and CA199 showed good diagnostic accuracy in predicting EC in symptomatic women [26]. The study of blood chemical markers and genotypic variables could increase the predictive power of the various models to have a clinical impact.

The limitations of the present study include (i) its retrospective nature; (ii) some independent variables of importance may be missing. Endometrial ultrasound characterization could have a predictive weight; (iii) the reproducibility of endometrial hyperplasia is poor among pathologists. However, the latest atypical and non-atypical classifications can mitigate this limitation [14,15].

The study’s strengths include the appropriate sample size of EC cases in relation to the number of variables studied. Endometrial sampling methods included 85% of women undergoing hysteroscopy, minimizing blind biopsies. It should be noted that our study had several referral cancer centers to make the results more generalizable.

## 5. Conclusions

The study of patient characteristics for predicting EC in women with AEH showed disappointing performance using regressions and ANNs. Although some variables were recurrent in EC, they likely do not contain good/optimal discriminating information. So, a risk stratification of AEH based on patient characteristics can be misleading in clinical practice. As the first predictive models in this area, they could be regarded as the starting point for future models that should include other individual factors (e.g., genotypic variables) to move towards more personalized medicine.

## Figures and Tables

**Table 1:** Patient characteristics.

Independent Variables	*n* (%) (sample Size = 629)
Age (median and interquartile ranges)	56.0 (51.0–65.0)
Menopause	427 (67.9)
Nulligravid	118 (18.8)
Smoking habit	121 (19.2)
Body Mass Index (reported as continuous variable; median and interquartile ranges)	27.0 (24.0–33.0)
Comorbidity	
Diabetes	18 (2.9)
Hypertension	192 (30.5)
Diabetes + Hypertension	43 (6.8)
Lynch Syndrome	9 (1.4)
Previous breast cancer	92 (14.6)
Previous Tamoxifen therapy	36 (5.7)
Hormonal therapy	
OC	33 (5.2)
HRT	17 (2.7)
Abnormal uterine bleeding	429 (68.2)
Endometrial sampling methods	
HSC-bio	383 (60.9)
HSC-res	153 (24.3)
D&C	93 (14.8)
Endometrial Cancer	193 (30.7)

D&C: dilation and curettage; HSC-bio: hysteroscopically guided biopsy; HSC-res: hysteroscopic endometrial resection; OC: oral contraceptive; HRT: hormonal replacement therapy.

**Table 2:** Univariate analysis comparing women with or without endometrial cancer at hysterectomy.

Women with Pre-Operative AEH	Final Histology (Hysterectomy)		
Independent Variables	No Endometrial Cancer (436) *n* (%)	Endometrial Cancer (193) *n* (%)	*p* Value
Age (median and interquartile ranges)	56 (51.0–64.0)	59 (52.0–69.0)	<0.001
Menopause	284 (65.1)	143 (74.1)	0.026
Nulligravid	77 (17.7)	41 (21.2)	0.288
Smoking habit	89 (20.4)	32 (16.6)	0.261
Body Mass Index (median and interquartile ranges)	27 (23.0–32.0)	29 (25.0–35.0)	<0.001
Comorbidity			0.311
Diabetes	14 (3.2)	4 (2.1)	
Hypertension	126 (28.9)	66 (34.2)	
Diabetes + Hypertension	27 (6.2)	16 (8.3)	
Lynch Syndromes	2 (0.5)	7 (3.6)	0.002
Previous breast cancer	73 (16.7)	19 (9.8)	0.024
Previous Tamoxifen therapy	29 (6.7)	7 (3.6)	0.132
Hormonal therapy users			0.273
OC	27 (6.2)	6 (3.1)	
HRT	12 (2.8)	5 (2.6)	
Abnormal uterine bleeding	291 (66.7)	138 (71.5)	0.237
Endometrial sampling methods			0.109
HSC-bio	260 (59.6)	123 (63.7)	
HSC-res	116 (26.6)	37 (19.2)	
D&C	60 (13.8)	33 (17.1)	

AEH: atypical endometrial hyperplasia; OC: oral contraceptive; HRT: hormonal replacement therapy. D&C: dilation and curettage; HSC-bio: hysteroscopically guided biopsy; HSC-res: hysteroscopic endometrial resection.

**Table 3:** Results of the quadratic regression performed on all number and variable combinations.

10-Fold Cross-Validation and Quadratic Regression	Train			Test			More Discriminating Variables	Explicit Variables
Variable Number (Number of Tested Combinations)	Mean Sensitivity	Mean Specificity	Mean AUC	Mean Sensitivity	Mean Specificity	Mean AUC		
2 (120)	55	64.2	0.625 (CI 95%: 0.593–0.656)	50.2	64.1	0.599 (CI 95%: 0.562–0.637)	1, 8	Age, BMI
	47.2	68.1	0.603 (CI 95%: 0.577–0.629)	46.1	68.8	0.588 (CI 95%: 0.542–0.633)	8, 13	BMI, BC
	40.4	70.4	0.571 (CI 95%: 0.567–0.575)	40.9	70.5	0.576 (CI 95%: 0.538–0.615)	6, 13	Hyp, BC
	65.3	48.4	0.575 (CI 95%: 0.567–0.582)	64.4	48.8	0.572 (CI 95%: 0.505–0.639)	2, 13	Mp, BC
	44	69.7	0.596 (CI 95%: 0.559–0.633)	42.4	69.5	0.57 (CI 95%: 0.515–0.625)	8, 15	BMI, RB
3 (560)	62.4	61.1	0.656 (CI 95%: 0.649–0.663)	62.1	60.5	0.643 (CI 95%: 0.593–0.692)	1, 8, 9	Age, BMI, Lynch
	59	61.6	0.653 (CI 95%: 0.646–0.659)	59.5	61.4	0.625 (CI 95%: 0.578–0.672)	1, 8, 13	Age, BMI, BC
	61.5	59.6	0.643 (CI 95%: 0.637–0.649)	61.7	59	0.62 (CI 95%: 0.574–0.666)	1, 8, 11	Age, BMI, OC
	53.8	64.4	0.63 (CI 95%: 0.597–0.664)	53.5	64.8	0.62 (CI 95%: 0.559–0.681)	1, 7, 8	Age, Dia, BMI
	60.9	59.7	0.642 (CI 95%: 0.635–0.649)	60.1	58.8	0.617 (CI 95%: 0.567–0.668)	1, 8, 10	Age, BMI, Tam
4 (1820)	61.4	62.8	0.671 (CI 95%: 0.662–0.68)	61.1	61.7	0.655 (CI 95%: 0.571–0.739)	1, 8, 9, 13	Age, BMI, Lynch, BC
	62.5	60.8	0.661 (CI 95%: 0.652–0.67)	61.8	60	0.649 (CI 95%: 0.56–0.737)	1, 8, 9, 11	Age, BMI, Lynch, OC
	62.7	60.8	0.66 (CI 95%: 0.652–0.669)	60.5	58.8	0.643 (CI 95%: 0.567–0.72)	1, 8, 9, 10	Age, BMI, Lynch, Tam
	61.7	62.2	0.666 (CI 95%: 0.658–0.675)	59.8	61.8	0.643 (CI 95%: 0.56–0.725)	1, 8, 9, 15	Age, BMI, Lynch, RB
	62	61.8	0.657 (CI 95%: 0.647–0.666)	60.5	60.7	0.64 (CI 95%: 0.55–0.731)	1, 8, 9, 12	Age, BMI, Lynch, TOS
5 (4368)	62.3	61.9	0.682 (CI 95%: 0.676–0.688)	60.1	61.8	0.649 (CI 95%: 0.608–0.691)	1, 7, 8, 9, 13	Age, Dia, BMI, Lynch, BC
	63	61.1	0.671 (CI 95%: 0.666–0.676)	61.5	60.1	0.642 (CI 95%: 0.597–0.686)	1, 7, 8, 10, 13	Age, Dia, BMI, Tam, BC
	62.8	62.5	0.674 (CI 95%: 0.668–0.68)	59.2	63	0.642 (CI 95%: 0.602–0.681)	1, 8, 9, 10, 13	Age, BMI, Lynch, Tam, BC
	62.5	60.4	0.67 (CI 95%: 0.665–0.676)	61.3	60.2	0.641 (CI 95%: 0.596–0.686)	1, 7, 8, 11, 13	Age, Dia, BMI, OC, BC
	60.7	64.8	0.672 (CI 95%: 0.665–0.678)	59	63	0.639 (CI 95%: 0.587–0.692)	1, 5, 7, 8, 13	Age, Bleed, Dia, BMI, BC
6 (8008)	59.7	66.3	0.685 (CI 95%: 0.678–0.691)	52.4	64.2	0.638 (CI 95%: 0.572–0.705)	1, 5, 8, 9, 11, 13	Age, Bleed, BMI, Lynch, OC, BC
	65.2	61	0.688 (CI 95%: 0.682–0.693)	61.1	59.3	0.638 (CI 95%: 0.586–0.69)	1, 2, 7, 8, 9, 13	Age, Mp, Dia, BMI, Lynch, BC
	64.3	64.6	0.69 (CI 95%: 0.683–0.697)	57.2	62.3	0.637 (CI 95%: 0.584–0.691)	1, 6, 8, 9, 13, 14	Age, Hyp, BMI, Lynch, BC, HB
	62.9	61.6	0.687 (CI 95%: 0.682–0.692)	58	61.5	0.637 (CI 95%: 0.589–0.685)	1, 7, 8, 9, 11, 13	Age, Dia, BMI, Lynch, OC, BC
	66.1	63.9	0.693 (CI 95%: 0.687–0.699)	59.5	64.5	0.633 (CI 95%: 0.584–0.682)	1, 7, 8, 9, 13, 14	Age, Dia, BMI, Lynch, BC, HB
7 (11,440)	68.5	60.1	0.694 (CI 95%: 0.688–0.701)	64.2	58.8	0.653 (CI 95%: 0.592–0.714)	1, 2, 7, 8, 9, 10, 13	Age, Mp, Dia, BMI, Lynch, Tam, BC
	65.1	61.6	0.692 (CI 95%: 0.685–0.699)	62.3	58.8	0.653 (CI 95%: 0.597–0.709)	1, 7, 8, 9, 10, 11, 13	Age, Dia, BMI, Lynch, Tam, OC, BC
	64.2	62.5	0.693 (CI 95%: 0.687–0.699)	61.4	59.9	0.646 (CI 95%: 0.59–0.701)	1, 7, 8, 9, 10, 12, 13	Age, Dia, BMI, Lynch, Tam, TOS, BC
	63.8	64.3	0.694 (CI 95%: 0.687–0.701)	59.4	62.2	0.643 (CI 95%: 0.588–0.698)	1, 5, 7, 8, 9, 10, 13	Age, Bleed, Dia, BMI, Lynch, Tam, BC
	65.9	60.9	0.691 (CI 95%: 0.686–0.697)	60.2	59.8	0.643 (CI 95%: 0.587–0.699)	1, 2, 7, 8, 9, 11, 13	Age, Mp, Dia, BMI, Lynch, OC, BC
8 (12,870)	69.8	61.7	0.711 (CI 95%: 0.705–0.718)	64.6	59	0.655 (CI 95%: 0.601–0.709)	1, 7, 8, 9, 10, 11, 13, 14	Age, Dia, BMI, Lynch, Tam, OC, BC, HB
	69.9	63.9	0.72 (CI 95%: 0.713–0.727)	66.8	59.7	0.654 (CI 95%: 0.599–0.709)	1, 6, 7, 8, 9, 10, 13, 14	Age, Hyp, Dia, BMI, Lynch, Tam, BC, HB
	67.8	64.4	0.715 (CI 95%: 0.709–0.722)	63.9	61.4	0.65 (CI 95%: 0.595–0.706)	1, 6, 7, 8, 9, 11, 13, 14	Age, Hyp, Dia, BMI, Lynch, OC, BC, HB
	69.2	62.7	0.709 (CI 95%: 0.703–0.715)	64.3	58.8	0.646 (CI 95%: 0.598–0.693)	1, 7, 8, 9, 10, 12, 13, 14	Age, Dia, BMI, Lynch, Tam, TOS, BC, HB
	63.9	62.5	0.698 (CI 95%: 0.69–0.705)	61.9	61	0.644 (CI 95%: 0.6–0.688)	1, 7, 8, 9, 10, 11, 12, 13	Age, Dia, BMI, Lynch, Tam, OC, TOS, BC
9 (11,440)	70.4	63	0.725 (CI 95%: 0.72–0.73)	64.1	60.1	0.655 (CI 95%: 0.606–0.704)	1, 3, 7, 8, 9, 10, 11, 13, 15	Age, Preg, Dia, BMI, Lynch, Tam, OC, BC, RB
	69.6	66.3	0.726 (CI 95%: 0.72–0.732)	63.6	63.9	0.649 (CI 95%: 0.596–0.702)	1, 3, 4, 7, 8, 9, 11, 13, 15	Age, Preg, Smoke, Dia, BMI, Lynch, OC, BC, RB
	69.1	62.8	0.715 (CI 95%: 0.709–0.72)	63.9	61.2	0.646 (CI 95%: 0.588–0.704)	1, 7, 8, 9, 10, 13, 14, 15, 16	Age, Dia, BMI, Lynch, Tam, BC, HB, RB, D&C
	70.2	63.6	0.716 (CI 95%: 0.711–0.722)	62.3	61.6	0.645 (CI 95%: 0.59–0.701)	1, 7, 8, 9, 11, 13, 14, 15, 16	Age, Dia, BMI, Lynch, OC, BC, HB, RB, D&C
	72.2	62.5	0.725 (CI 95%: 0.72–0.729)	64.9	59.7	0.645 (CI 95%: 0.594–0.696)	1, 7, 8, 9, 10, 11, 13, 14, 15	Age, Dia, BMI, Lynch, Tam, OC, BC, HB, RB
10 (8008)	67.8	68.3	0.738 (CI 95%: 0.735–0.742)	55.6	63.5	0.627 (CI 95%: 0.585–0.669)	1, 3, 4, 5, 7, 8, 9, 11, 13, 15	Age, Preg, Smoke, Bleed, Dia, BMI, Lynch, OC, BC, RB
	74.3	60.7	0.734 (CI 95%: 0.732–0.737)	67.1	57	0.626 (CI 95%: 0.594–0.658)	1, 2, 6, 7, 8, 9, 10, 12, 13, 14	Age, Mp, Hyp, Dia, BMI, Lynch, Tam, TOS, BC, HB
	69.5	64.5	0.733 (CI 95%: 0.729–0.736)	57.1	60.2	0.626 (CI 95%: 0.574–0.677)	1, 6, 7, 8, 9, 11, 13, 14, 15, 16	Age, Hyp, Dia, BMI, Lynch, OC, BC, HB, RB, D&C
	72.5	62.3	0.733 (CI 95%: 0.732–0.735)	61.4	59.4	0.624 (CI 95%: 0.592–0.657)	1, 6, 7, 8, 9, 10, 11, 12, 13, 15	Age, Hyp, Dia, BMI, Lynch, Tam, OC, TOS, BC, RB
	69.7	63	0.718 (CI 95%: 0.715–0.72)	60.3	61	0.624 (CI 95%: 0.585–0.662)	1, 4, 6, 7, 8, 9, 10, 11, 12, 13	Age, Smoke, Hyp, Dia, BMI, Lynch, Tam, OC, TOS, BC
11 (4368)	71.3	64.6	0.739 (CI 95%: 0.733–0.745)	64.5	59.2	0.642 (CI 95%: 0.598–0.686)	1, 6, 7, 8, 9, 10, 12, 13, 14, 15, 16	Age, Hyp, Dia, BMI, Lynch, Tam, TOS, BC, HB, RB, D&C
	73.5	62	0.74 (CI 95%: 0.734–0.746)	64.7	56.7	0.641 (CI 95%: 0.596–0.687)	1, 2, 6, 7, 8, 9, 10, 13, 14, 15, 16	Age, Mp, Hyp, Dia, BMI, Lynch, Tam, BC, HB, RB, D&C
	70.9	64.7	0.747 (CI 95%: 0.742–0.752)	63.8	57.3	0.638 (CI 95%: 0.592–0.685)	1, 2, 3, 6, 7, 8, 9, 10, 12, 13, 14	Age, Mp, Preg, Hyp, Dia, BMI, Lynch, Tam, TOS, BC, HB
	70.4	65.1	0.732 (CI 95%: 0.726–0.737)	60.6	62.3	0.634 (CI 95%: 0.577–0.691)	1, 4, 7, 8, 9, 11, 12, 13, 14, 15, 16	Age, Smoke, Dia, BMI, Lynch, OC, TOS, BC, HB, RB, D&C
	71.4	64.8	0.742 (CI 95%: 0.737–0.747)	60.8	58.5	0.634 (CI 95%: 0.595–0.673)	1, 6, 7, 8, 9, 10, 11, 13, 14, 15, 16	Age, Hyp, Dia, BMI, Lynch, Tam, OC, BC, HB, RB, D&C
12 (1820)	76.3	60.9	0.741 (CI 95%: 0.735–0.747)	64.7	54.8	0.607 (CI 95%: 0.544–0.669)	1, 2, 4, 7, 8, 9, 10, 11, 13, 14, 15, 16	Age, Mp, Smoke, Dia, BMI, Lynch, Tam, OC, BC, HB, RB, D&C
	75.2	61.6	0.759 (CI 95%: 0.752–0.765)	63.1	53.7	0.606 (CI 95%: 0.546–0.666)	1, 2, 4, 6, 7, 8, 9, 10, 11, 13, 14, 15	Age, Mp, Smoke, Hyp, Dia, BMI, Lynch, Tam, OC, BC, HB, RB
	75.2	61.6	0.759 (CI 95%: 0.752–0.765)	63.1	53.7	0.606 (CI 95%: 0.545–0.666)	1, 2, 4, 6, 7, 8, 9, 10, 11, 13, 14, 16	Age, Mp, Smoke, Hyp, Dia, BMI, Lynch, Tam, OC, BC, HB, D&C
	73.7	64.1	0.744 (CI 95%: 0.74–0.747)	59.2	59.6	0.606 (CI 95%: 0.574–0.637)	1, 2, 3, 5, 7, 8, 9, 10, 13, 14, 15, 16	Age, Mp, Preg, Bleed, Dia, BMI, Lynch, Tam, BC, HB, RB, D&C
	72.5	65.6	0.753 (CI 95%: 0.747–0.758)	57.8	59.1	0.605 (CI 95%: 0.563–0.648)	1, 2, 3, 5, 6, 7, 8, 10, 13, 14, 15, 16	Age, Mp, Preg, Bleed, Hyp, Dia, BMI, Tam, BC, HB, RB, D&C
13 (560)	71.2	65.4	0.762 (CI 95%: 0.757–0.766)	59.6	59.5	0.619 (CI 95%: 0.572–0.667)	1, 2, 3, 6, 7, 8, 9, 10, 12, 13, 14, 15, 16	Age, Mp, Preg, Hyp, Dia, BMI, Lynch, Tam, TOS, BC, HB, RB, D&C
	72.2	64.9	0.762 (CI 95%: 0.758–0.765)	57.3	58.8	0.614 (CI 95%: 0.572–0.657)	1, 2, 3, 6, 7, 8, 9, 10, 11, 13, 14, 15, 16	Age, Mp, Preg, Hyp, Dia, BMI, Lynch, Tam, OC, BC, HB, RB, D&C
	71.2	66.4	0.756 (CI 95%: 0.751–0.761)	57.1	61.1	0.613 (CI 95%: 0.567–0.66)	1, 3, 4, 5, 7, 8, 9, 11, 12, 13, 14, 15, 16	Age, Preg, Smoke, Bleed, Dia, BMI, Lynch, OC, TOS, BC, HB, RB, D&C
	71.5	65.1	0.759 (CI 95%: 0.755–0.762)	58.6	58.8	0.613 (CI 95%: 0.576–0.649)	1, 3, 6, 7, 8, 9, 10, 11, 12, 13, 14, 15, 16	Age, Preg, Hyp, Dia, BMI, Lynch, Tam, OC, TOS, BC, HB, RB, D&C
	75.2	61.8	0.759 (CI 95%: 0.755–0.763)	62.9	54.6	0.612 (CI 95%: 0.55–0.675)	1, 2, 4, 6, 7, 8, 9, 10, 11, 13, 14, 15, 16	Age, Mp, Smoke, Hyp, Dia, BMI, Lynch, Tam, OC, BC, HB, RB, D&C
14 (120)	73.5	66.7	0.776 (CI 95%: 0.77–0.782)	56.6	60.1	0.621 (CI 95%: 0.559–0.683)	1, 2, 3, 4, 5, 6, 7, 8, 9, 11, 13, 14, 15, 16	Age, Mp, Preg, Smoke, Bleed, Hyp, Dia, BMI, Lynch, OC, BC, HB, RB, D&C
	73.8	66.7	0.775 (CI 95%: 0.768–0.782)	55	60.5	0.62 (CI 95%: 0.569–0.671)	1, 2, 3, 5, 6, 7, 8, 9, 10, 11, 13, 14, 15, 16	Age, Mp, Preg, Bleed, Hyp, Dia, BMI, Lynch, Tam, OC, BC, HB, RB, D&C
	73.6	67.3	0.777 (CI 95%: 0.769–0.784)	53.8	60.8	0.62 (CI 95%: 0.56–0.679)	1, 2, 3, 4, 5, 6, 7, 8, 9, 10, 13, 14, 15, 16	Age, Mp, Preg, Smoke, Bleed, Hyp, Dia, BMI, Lynch, Tam, BC, HB, RB, D&C
	72.8	66.4	0.766 (CI 95%: 0.761–0.772)	58.4	59.2	0.615 (CI 95%: 0.557–0.673)	1, 3, 4, 5, 7, 8, 9, 10, 11, 12, 13, 14, 15, 16	Age, Preg, Smoke, Bleed, Dia, BMI, Lynch, Tam, OC, TOS, BC, HB, RB, D&C
	74.3	67.2	0.774 (CI 95%: 0.768–0.781)	56.7	61	0.614 (CI 95%: 0.558–0.67)	1, 2, 3, 5, 6, 7, 8, 9, 10, 12, 13, 14, 15, 16	Age, Mp, Preg, Bleed, Hyp, Dia, BMI, Lynch, Tam, TOS, BC, HB, RB, D&C
15 (16)	74.7	67.1	0.783 (CI 95%: 0.777–0.79)	57.1	57.3	0.59 (CI 95%: 0.54–0.641)	1, 2, 3, 5, 6, 7, 8, 9, 10, 11, 12, 13, 14, 15, 16	Age, Mp, Preg, Bleed, Hyp, Dia, BMI, Lynch, Tam, OC, TOS, BC, HB, RB, D&C
	74.6	66.4	0.788 (CI 95%: 0.781–0.794)	57.9	53.9	0.588 (CI 95%: 0.538–0.638)	1, 2, 3, 4, 5, 6, 7, 8, 9, 10, 11, 13, 14, 15, 16	Age, Mp, Preg, Smoke, Bleed, Hyp, Dia, BMI, Lynch, Tam, OC, BC, HB, RB, D&C
	74.6	68.3	0.787 (CI 95%: 0.78–0.794)	59.5	56.4	0.588 (CI 95%: 0.532–0.644)	1, 2, 3, 4, 5, 6, 7, 8, 9, 10, 12, 13, 14, 15, 16	Age, Mp, Preg, Smoke, Bleed, Hyp, Dia, BMI, Lynch, Tam, TOS, BC, HB, RB, D&C
	74.9	67.6	0.786 (CI 95%: 0.781–0.791)	56.9	55.1	0.587 (CI 95%: 0.538–0.635)	1, 2, 3, 4, 5, 6, 7, 8, 9, 11, 12, 13, 14, 15, 16	Age, Mp, Preg, Smoke, Bleed, Hyp, Dia, BMI, Lynch, OC, TOS, BC, HB, RB, D&C
	76.3	65.1	0.785 (CI 95%: 0.779–0.792)	60.6	53	0.584 (CI 95%: 0.54–0.629)	1, 2, 3, 4, 5, 6, 7, 8, 10, 11, 12, 13, 14, 15, 16	Age, Mp, Preg, Smoke, Bleed, Hyp, Dia, BMI, Tam, OC, TOS, BC, HB, RB, D&C

1 Age; 2 Menopause (Mp); 3 previous pregnancies (Preg); 4 Smoking habit (Smoke); 5 abnormal uterine bleeding (bleed); 6 hypertension (Hyp); 7 diabet (Dia); 8 BMI; 9 hereditary Lynch syndrome (Lynch); 10 Previous Tamoxifen Therapy (Tam); 11 hormonal therapy with OC (OC); 12 hormonal therapy use TOS (TOS); 13 previous breast cancer (BC); 14 hysteroscopically guided biopsy (HB); 15 hysteroscopic endometrial resection (RB); 16 D&C dilation and curettage.

**Table 4:** Results of the Patternnet algorithm trained with the 10-fold cross-validation.

10-Fold Cross-Validation and 3*N Patternnet ANN	Train			Test			More Discriminating Variables	Explicit Variables
Variable Number (Number of Tested Combinations)	Mean Sensitivity	Mean Specificity	Mean AUC	Mean Sensitivity	Mean Specificity	Mean AUC		
2 (120)	52.9	65.3	0.636 (CI 95%: 0.626–0.646)	50.3	63.8	0.629 (CI 95%: 0.56–0.698)	1, 8	Age, BMI
	49.6	66.1	0.616 (CI 95%: 0.609–0.623)	50.3	65.9	0.613 (CI 95%: 0.544–0.682)	1, 14	Age, HB
	50.1	66.5	0.618 (CI 95%: 0.61–0.626)	49.4	66.1	0.61 (CI 95%: 0.546–0.674)	1, 9	Age, Lynch
	53.8	61.9	0.613 (CI 95%: 0.608–0.618)	52.1	61.9	0.61 (CI 95%: 0.562–0.657)	8, 13	BMI, BC
	63.5	53.2	0.618 (CI 95%: 0.61–0.626)	61.9	52.2	0.606 (CI 95%: 0.56–0.652)	8, 15	BMI, RB
3 (560)	66.4	53.7	0.64 (CI 95%: 0.616–0.664)	67.6	55.4	0.658 (CI 95%: 0.598–0.718)	2, 8, 14	Mp, BMI, HB
	60.7	61.4	0.647 (CI 95%: 0.636–0.659)	65.2	59.1	0.657 (CI 95%: 0.598–0.717)	1, 8, 9	Age, BMI, Lynch
	60.3	62	0.651 (CI 95%: 0.634–0.667)	62.9	62	0.648 (CI 95%: 0.571–0.726)	1, 8, 13	Age, BMI, BC
	50.8	67.5	0.641 (CI 95%: 0.631–0.65)	53	67	0.646 (CI 95%: 0.576–0.716)	1, 3, 8	Age, Preg, BMI
	60.2	62.5	0.641 (CI 95%: 0.627–0.656)	59.3	61.5	0.644 (CI 95%: 0.566–0.722)	1, 8, 15	Age, BMI, RB
4 (1820)	63.3	66	0.683 (CI 95%: 0.67–0.697)	65.8	66.5	0.691 (CI 95%: 0.657–0.725)	1, 8, 9, 14	Age, BMI, Lynch, HB
	68.1	61.6	0.689 (CI 95%: 0.683–0.694)	67.4	60	0.688 (CI 95%: 0.653–0.722)	1, 5, 8, 14	Age, Bleed, BMI, HB
	62.7	62.4	0.671 (CI 95%: 0.666–0.677)	63.1	62.2	0.671 (CI 95%: 0.646–0.697)	1, 8, 9, 15	Age, BMI, Lynch, RB
	67.8	59.2	0.674 (CI 95%: 0.659–0.69)	66.3	62.8	0.668 (CI 95%: 0.617–0.72)	1, 2, 8, 14	Age, Mp, BMI, HB
	63.6	59.4	0.658 (CI 95%: 0.654–0.663)	64.5	59.6	0.666 (CI 95%: 0.626–0.706)	1, 3, 8, 9	Age, Preg, BMI, Lynch
5 (4368)	65.8	65	0.695 (CI 95%: 0.689–0.702)	67.8	63.4	0.702 (CI 95%: 0.656–0.749)	1, 2, 8, 13, 14	Age, Mp, BMI, BC, HB
	63.6	63.5	0.692 (CI 95%: 0.688–0.697)	66	63.6	0.701 (CI 95%: 0.665–0.737)	1, 8, 9, 13, 15	Age, BMI, Lynch, BC, RB
	63.9	63.6	0.682 (CI 95%: 0.675–0.689)	61.4	63.9	0.688 (CI 95%: 0.648–0.729)	1, 2, 8, 9, 14	Age, Mp, BMI, Lynch, HB
	63.2	62.5	0.668 (CI 95%: 0.659–0.678)	65.7	60.6	0.688 (CI 95%: 0.619–0.756)	1, 2, 6, 8, 14	Age, Mp, Hyp, BMI, HB
	65.4	61.7	0.683 (CI 95%: 0.675–0.69)	67.4	61.3	0.687 (CI 95%: 0.636–0.737)	1, 8, 9, 12, 14	Age, BMI, Lynch, TOS, HB
6 (8008)	68.4	60.8	0.705 (CI 95%: 0.7–0.711)	67.8	60.3	0.702 (CI 95%: 0.653–0.751)	1, 6, 7, 8, 14, 16	Age, Hyp, Dia, BMI, HB, D&C
	66.2	66.7	0.708 (CI 95%: 0.702–0.714)	64.8	66.4	0.696 (CI 95%: 0.64–0.752)	1, 7, 8, 9, 11, 14	Age, Dia, BMI, Lynch, OC, HB
	68.6	63.9	0.708 (CI 95%: 0.698–0.718)	67.4	63.5	0.694 (CI 95%: 0.609–0.779)	1, 2, 7, 8, 9, 14	Age, Mp, Dia, BMI, Lynch, HB
	67.1	61.5	0.699 (CI 95%: 0.693–0.704)	65.8	61.1	0.693 (CI 95%: 0.643–0.744)	1, 5, 8, 9, 11, 15	Age, Bleed, BMI, Lynch, OC, RB
	72.6	62.6	0.716 (CI 95%: 0.701–0.731)	67.2	62.6	0.693 (CI 95%: 0.633–0.753)	1, 2, 8, 9, 13, 14	Age, Mp, BMI, Lynch, BC, HB
7 (11,440)	69.9	62.5	0.707 (CI 95%: 0.687–0.728)	70.3	62	0.71 (CI 95%: 0.667–0.753)	1, 8, 9, 11, 13, 15, 16	Age, BMI, Lynch, OC, BC, RB, D&C
	66.4	67.1	0.717 (CI 95%: 0.712–0.722)	66	67.5	0.709 (CI 95%: 0.655–0.764)	1, 2, 3, 4, 6, 8, 14	Age, Mp, Preg, Smoke, Hyp, BMI, HB
	66	63.5	0.7 (CI 95%: 0.692–0.708)	68	64.7	0.705 (CI 95%: 0.667–0.744)	1, 3, 7, 8, 9, 13, 14	Age, Preg, Dia, BMI, Lynch, BC, HB
	60.3	68.5	0.699 (CI 95%: 0.696–0.702)	59.7	68.8	0.703 (CI 95%: 0.677–0.728)	1, 3, 5, 6, 8, 9, 13	Age, Preg, Bleed, Hyp, BMI, Lynch, BC
	67.1	61.2	0.707 (CI 95%: 0.694–0.721)	65.1	62.2	0.701 (CI 95%: 0.648–0.755)	1, 6, 8, 9, 10, 13, 15	Age, Hyp, BMI, Lynch, Tam, BC, RB
8 (12,870)	67.1	67.2	0.738 (CI 95%: 0.696–0.781)	66.5	65.3	0.736 (CI 95%: 0.643–0.828)	1, 2, 6, 7, 8, 9, 10, 14	Age, Mp, Hyp, Dia, BMI, Lynch, Tam, HB
	67	65.5	0.733 (CI 95%: 0.728–0.739)	67.5	66.3	0.731 (CI 95%: 0.677–0.786)	1, 2, 4, 6, 8, 13, 14, 16	Age, Mp, Smoke, Hyp, BMI, BC, HB, D&C
	70.9	63.6	0.733 (CI 95%: 0.726–0.74)	70.1	63.5	0.729 (CI 95%: 0.662–0.797)	1, 2, 5, 6, 8, 9, 10, 14	Age, Mp, Bleed, Hyp, BMI, Lynch, Tam, HB
	68.2	63.8	0.721 (CI 95%: 0.716–0.726)	67.5	64.8	0.72 (CI 95%: 0.67–0.771)	1, 4, 6, 7, 8, 9, 13, 15	Age, Smoke, Hyp, Dia, BMI, Lynch, BC, RB
	72.8	60.6	0.717 (CI 95%: 0.713–0.721)	71.3	60.1	0.719 (CI 95%: 0.675–0.763)	1, 5, 8, 9, 10, 12, 15, 16	Age, Bleed, BMI, Lynch, Tam, TOS, RB, D&C
9 (11,440)	69.1	66.2	0.731 (CI 95%: 0.727–0.735)	67.5	67.5	0.722 (CI 95%: 0.672–0.773)	1, 5, 6, 7, 8, 9, 12, 13, 15	Age, Bleed, Hyp, Dia, BMI, Lynch, TOS, BC, RB
	66.8	67.1	0.741 (CI 95%: 0.735–0.746)	63.9	68.2	0.721 (CI 95%: 0.65–0.793)	1, 3, 6, 8, 9, 10, 13, 15, 16	Age, Preg, Hyp, BMI, Lynch, Tam, BC, RB, D&C
	68.7	66.1	0.732 (CI 95%: 0.727–0.737)	67.4	66.8	0.72 (CI 95%: 0.663–0.778)	1, 3, 4, 5, 6, 7, 8, 9, 14	Age, Preg, Smoke, Bleed, Hyp, Dia, BMI, Lynch, HB
	66.2	62.4	0.721 (CI 95%: 0.718–0.725)	66.1	62.7	0.72 (CI 95%: 0.677–0.763)	1, 3, 6, 7, 8, 10, 11, 14, 16	Age, Preg, Hyp, Dia, BMI, Tam, OC, HB, D&C
	71.8	62.1	0.729 (CI 95%: 0.711–0.747)	73.6	61	0.717 (CI 95%: 0.651–0.783)	2, 4, 5, 6, 7, 8, 10, 14, 15	Mp, Smoke, Bleed, Hyp, Dia, BMI, Tam, HB, RB
10 (8008)	69.2	67	0.754 (CI 95%: 0.747–0.761)	68.4	67.1	0.752 (CI 95%: 0.681–0.824)	1, 4, 6, 7, 8, 9, 12, 13, 14, 15	Age, Smoke, Hyp, Dia, BMI, Lynch, TOS, BC, HB, RB
	68.5	66.3	0.743 (CI 95%: 0.739–0.748)	69.6	65.1	0.749 (CI 95%: 0.709–0.789)	1, 4, 6, 7, 8, 10, 11, 13, 14, 16	Age, Smoke, Hyp, Dia, BMI, Tam, OC, BC, HB, D&C
	70.9	66.3	0.753 (CI 95%: 0.74–0.765)	72.6	65.2	0.745 (CI 95%: 0.686–0.804)	1, 3, 5, 6, 8, 9, 10, 11, 12, 14	Age, Preg, Bleed, Hyp, BMI, Lynch, Tam, OC, TOS, HB
	75.5	58.5	0.736 (CI 95%: 0.732–0.741)	76	58.8	0.738 (CI 95%: 0.685–0.791)	1, 3, 5, 7, 8, 9, 11, 13, 14, 15	Age, Preg, Bleed, Dia, BMI, Lynch, OC, BC, HB, RB
	67.2	65.8	0.74 (CI 95%: 0.734–0.745)	66	66.7	0.735 (CI 95%: 0.671–0.798)	1, 6, 7, 8, 9, 11, 12, 13, 14, 15	Age, Hyp, Dia, BMI, Lynch, OC, TOS, BC, HB, RB
11 (4368)	68.9	64.9	0.749 (CI 95%: 0.744–0.754)	68.3	65.7	0.752 (CI 95%: 0.7–0.805)	1, 3, 4, 5, 6, 8, 9, 11, 13, 15, 16	Age, Preg, Smoke, Bleed, Hyp, BMI, Lynch, OC, BC, RB, D&C
	72.5	64.6	0.756 (CI 95%: 0.752–0.76)	71.6	65.7	0.75 (CI 95%: 0.703–0.797)	1, 3, 4, 5, 6, 7, 8, 10, 11, 12, 14	Age, Preg, Smoke, Bleed, Hyp, Dia, BMI, Tam, OC, TOS, HB
	71.5	64.6	0.74 (CI 95%: 0.73–0.75)	69.6	64.1	0.744 (CI 95%: 0.667–0.82)	1, 4, 5, 6, 7, 8, 9, 11, 13, 14, 16	Age, Smoke, Bleed, Hyp, Dia, BMI, Lynch, OC, BC, HB, D&C
	67.2	67.5	0.746 (CI 95%: 0.738–0.753)	65.3	67.9	0.742 (CI 95%: 0.678–0.807)	1, 3, 6, 7, 8, 9, 10, 12, 13, 14, 15	Age, Preg, Hyp, Dia, BMI, Lynch, Tam, TOS, BC, HB, RB
	75.1	59.7	0.748 (CI 95%: 0.744–0.753)	73.8	58.5	0.74 (CI 95%: 0.684–0.796)	1, 2, 4, 6, 7, 8, 11, 12, 14, 15, 16	Age, Mp, Smoke, Hyp, Dia, BMI, OC, TOS, HB, RB, D&C
12 (1820)	67.1	68.6	0.753 (CI 95%: 0.747–0.758)	68.1	68.9	0.76 (CI 95%: 0.706–0.814)	1, 3, 4, 5, 6, 7, 8, 9, 12, 13, 15, 16	Age, Preg, Smoke, Bleed, Hyp, Dia, BMI, Lynch, TOS, BC, RB, D&C
	73.3	66.2	0.755 (CI 95%: 0.747–0.764)	72.6	65.8	0.754 (CI 95%: 0.681–0.826)	1, 2, 3, 4, 5, 6, 7, 8, 10, 13, 14, 16	Age, Mp, Preg, Smoke, Bleed, Hyp, Dia, BMI, Tam, BC, HB, D&C
	68.4	67.5	0.751 (CI 95%: 0.744–0.758)	68.4	66.9	0.753 (CI 95%: 0.695–0.812)	1, 2, 3, 4, 6, 7, 8, 9, 11, 12, 15, 16	Age, Mp, Preg, Smoke, Hyp, Dia, BMI, Lynch, OC, TOS, RB, D&C
	64.4	69.4	0.747 (CI 95%: 0.74–0.754)	67.4	69.8	0.753 (CI 95%: 0.701–0.805)	1, 2, 3, 4, 5, 6, 8, 9, 10, 13, 14, 15	Age, Mp, Preg, Smoke, Bleed, Hyp, BMI, Lynch, Tam, BC, HB, RB
	73.4	61.6	0.741 (CI 95%: 0.735–0.747)	73.2	60.8	0.748 (CI 95%: 0.689–0.808)	1, 2, 3, 4, 6, 7, 8, 9, 11, 14, 15, 16	Age, Mp, Preg, Smoke, Hyp, Dia, BMI, Lynch, OC, HB, RB, D&C
13 (560)	73.2	66.2	0.765 (CI 95%: 0.76–0.771)	71.7	65.5	0.756 (CI 95%: 0.707–0.805)	1, 5, 6, 7, 8, 9, 10, 11, 12, 13, 14, 15, 16	Age, Bleed, Hyp, Dia, BMI, Lynch, Tam, OC, TOS, BC, HB, RB, D&C
	73.3	60.2	0.739 (CI 95%: 0.729–0.748)	75.4	60.8	0.742 (CI 95%: 0.669–0.816)	1, 2, 3, 4, 6, 7, 8, 9, 10, 11, 13, 14, 15	Age, Mp, Preg, Smoke, Hyp, Dia, BMI, Lynch, Tam, OC, BC, HB, RB
	72.2	65.9	0.749 (CI 95%: 0.743–0.755)	72.2	64.2	0.741 (CI 95%: 0.68–0.802)	1, 2, 3, 4, 5, 6, 7, 8, 9, 13, 14, 15, 16	Age, Mp, Preg, Smoke, Bleed, Hyp, Dia, BMI, Lynch, BC, HB, RB, D&C
	71.2	65	0.746 (CI 95%: 0.732–0.761)	69.5	63.8	0.738 (CI 95%: 0.639–0.837)	1, 2, 3, 4, 5, 6, 7, 8, 10, 13, 14, 15, 16	Age, Mp, Preg, Smoke, Bleed, Hyp, Dia, BMI, Tam, BC, HB, RB, D&C
	70.7	66	0.74 (CI 95%: 0.73–0.749)	72.3	66.2	0.737 (CI 95%: 0.657–0.817)	1, 2, 3, 4, 6, 7, 8, 9, 11, 12, 13, 14, 15	Age, Mp, Preg, Smoke, Hyp, Dia, BMI, Lynch, OC, TOS, BC, HB, RB
14 (120)	67.8	66.1	0.73 (CI 95%: 0.727–0.733)	68.5	65.1	0.739 (CI 95%: 0.71–0.767)	1, 2, 3, 4, 6, 7, 8, 9, 11, 12, 13, 14, 15, 16	Age, Mp, Preg, Smoke, Hyp, Dia, BMI, Lynch, OC, TOS, BC, HB, RB, D&C
	75.4	62.1	0.737 (CI 95%: 0.703–0.77)	72	62.6	0.727 (CI 95%: 0.649–0.804)	1, 2, 3, 4, 5, 6, 8, 9, 10, 11, 12, 14, 15, 16	Age, Mp, Preg, Smoke, Bleed, Hyp, BMI, Lynch, Tam, OC, TOS, HB, RB, D&C
	72.3	62.3	0.738 (CI 95%: 0.734–0.742)	71.5	63.6	0.726 (CI 95%: 0.681–0.77)	1, 3, 4, 5, 6, 7, 8, 9, 10, 12, 13, 14, 15, 16	Age, Preg, Smoke, Bleed, Hyp, Dia, BMI, Lynch, Tam, TOS, BC, HB, RB, D&C
	65.9	67.2	0.724 (CI 95%: 0.718–0.73)	65.9	66.7	0.723 (CI 95%: 0.661–0.786)	1, 2, 3, 4, 5, 6, 7, 8, 9, 10, 11, 12, 14, 16	Age, Mp, Preg, Smoke, Bleed, Hyp, Dia, BMI, Lynch, Tam, OC, TOS, HB, D&C
	67.3	66.7	0.72 (CI 95%: 0.715–0.724)	65.2	67.3	0.722 (CI 95%: 0.684–0.76)	1, 2, 3, 4, 5, 6, 7, 8, 10, 11, 13, 14, 15, 16	Age, Mp, Preg, Smoke, Bleed, Hyp, Dia, BMI, Tam, OC, BC, HB, RB, D&C
15 (16)	70.3	68	0.753 (CI 95%: 0.741–0.766)	70.4	67.7	0.743 (CI 95%: 0.642–0.844)	1, 2, 3, 4, 5, 6, 7, 8, 9, 10, 11, 12, 13, 14, 15	Age, Mp, Preg, Smoke, Bleed, Hyp, Dia, BMI, Lynch, Tam, OC, TOS, BC, HB, RB
	68.5	66.1	0.728 (CI 95%: 0.714–0.742)	66.4	64.9	0.713 (CI 95%: 0.629–0.796)	1, 2, 3, 4, 5, 6, 7, 8, 9, 11, 12, 13, 14, 15, 16	Age, Mp, Preg, Smoke, Bleed, Hyp, Dia, BMI, Lynch, OC, TOS, BC, HB, RB, D&C
	64.2	66	0.711 (CI 95%: 0.696–0.725)	65.9	65.9	0.708 (CI 95%: 0.629–0.786)	1, 2, 3, 4, 6, 7, 8, 9, 10, 11, 12, 13, 14, 15, 16	Age, Mp, Preg, Smoke, Hyp, Dia, BMI, Lynch, Tam, OC, TOS, BC, HB, RB, D&C
	69.1	62.4	0.699 (CI 95%: 0.694–0.704)	70.1	62	0.705 (CI 95%: 0.63–0.779)	1, 2, 3, 4, 5, 6, 8, 9, 10, 11, 12, 13, 14, 15, 16	Age, Mp, Preg, Smoke, Bleed, Hyp, BMI, Lynch, Tam, OC, TOS, BC, HB, RB, D&C
	66	64.8	0.729 (CI 95%: 0.719–0.739)	62.6	63.1	0.703 (CI 95%: 0.61–0.795)	1, 2, 3, 4, 5, 6, 7, 8, 9, 10, 11, 13, 14, 15, 16	Age, Mp, Preg, Smoke, Bleed, Hyp, Dia, BMI, Lynch, Tam, OC, BC, HB, RB, D&C

1 Age; 2 Menopause (Mp); 3 previous pregnancies (Preg); 4 Smoking habit (Smoke); 5 abnormal uterine bleeding (bleed); 6 hypertension (Hyp); 7 diabet (Dia); 8 BMI; 9 hereditary Lynch syndrome (Lynch); 10 Previous Tamoxifen therapy (Tam); 11 hormonal therapy with OC (OC); 12 hormonal therapy use TOS (TOS); 13 previous breast cancer (BC); 14 hysteroscopically guided biopsy (HB); 15 hysteroscopic endometrial resection (RB); 16 D&C dilation and curettage. For each reduced model having N variables, an artificial neural network having 3*N nodes was used. Thus, for the two-variable model, a network of six nodes was used. For three variables, nine nodes, and so on. The number of nodes n is variable and is shown in the first column of the table. Many configurations were tested with different nodes, but this was the one with the best performance and the least number of nodes.

**Table 5:** Comparison of recurrent patient characteristics in women with high- vs. no-high-risk EC.

Independent Variables	High-Risk Disease (26) *n* (%)	No High-Risk Disease (167) *n* (%)	*p* Value
Age (median and interquartile ranges)	64 (55.0–71.0)	58 (52.0–68.8)	0.313
Body Mass Index (median and interquartile ranges)	31 (26.0–34.0)	29 (25.0–35.0)	0.453
Comorbidity			0.337
Diabetes	1 (3.8)	3 (1.8)	
Hypertension	10 (38.5)	56 (33.5)	
Diabetes + Hypertension	4 (15.4)	12 (7.2)	
Lynch Syndromes	1 (3.8)	6 (3.6)	0.948
Previous breast cancer	3 (11.5)	16 (9.6)	0.755

## Data Availability

Data are available upon reasonable request.

## Supplementary Materials

The following supporting information can be downloaded at: https://www.mdpi.com/article/10.3390/cancers16010172/s1, Table S1: Scheme used to evaluate the performance of prediction models; Table S2: Surgical characteristics; Table S3: Results of the linear regression performed on all number and variable combinations; Table S4: Results from Support Vector Machine-based predictors; Figure S1: Comparative analysis of three different functions.

## References

[1] Gücer F., Reich O., Tamussino K., Bader A.A., Pieber D., Schöll W., Haas J., Petru E. (1998). Concomitant Endometrial Hyperplasia in Patients with Endometrial Carcinoma. Gynecol. Oncol..

[2] Widra E., Dunton C., McHugh M., Palazzo J. (1995). Endometrial hyperplasia and the risk of carcinoma. Int. J. Gynecol. Cancer.

[3] Whyte J.S., Gurney E.P., Curtin J.P., Blank S.V. (2010). Lymph node dissection in the surgical management of atypical endometrial hyperplasia. Am. J. Obstet. Gynecol..

[4] Touhami O., Grégoire J., Renaud M.-C., Sebastianelli A., Grondin K., Plante M. (2018). The utility of sentinel lymph node mapping in the management of endometrial atypical hyperplasia. Gynecol. Oncol..

[5] Kurman R.J., Kaminski P.F., Norris H.J. (1985). The behavior of endometrial hyperplasia. A long-term study of “untreated” hyperplasia in 170 patients. Cancer.

[6] Concin N., Matias-Guiu X., Vergote I., Cibula D., Mirza M.R., Marnitz S., Ledermann J.A., Bosse T., Chargari T., Fagotti A. (2021). ESGO/ESTRO/ESP guidelines for the management of patients with endometrial carcinoma. Int. J. Gynecol. Cancer.

[7] Shalowitz D.I., Goodwin A., Schoenbachler N. (2019). Does surgical treatment of atypical endometrial hyperplasia require referral to a gynecologic oncologist?. Am. J. Obstet. Gynecol..

[8] Wise M.R., Gill P., Lensen S., Thompson J.M., Farquhar C.M. (2016). Body mass index trumps age in decision for endometrial biopsy: Cohort study of symptomatic premenopausal women. Am. J. Obstet. Gynecol..

[9] Vetter M.H., Smith B., Benedict J., Hade E.M., Bixel K., Copeland L.J., Cohn D.E., Fowler J.M., O’malley D., Salani R. (2020). Preoperative predictors of endometrial cancer at time of hysterectomy for endometrial intraepithelial neoplasia or complex atypical hyperplasia. Am. J. Obstet. Gynecol..

[10] Matsuo K., Ramzan A.A., Gualtieri M.R., Mhawech-Fauceglia P., Machida H., Moeini A., Dancz C.E., Ueda Y., Roman L.D. (2015). Prediction of concurrent endometrial carcinoma in women with endometrial hyperplasia. Gynecol. Oncol..

[11] Giannella L., Carpini G.D., Sopracordevole F., Papiccio M., Serri M., Giorda G., Tsiroglou D., Del Fabro A., Ciavattini A. (2020). Atypical Endometrial Hyperplasia and Unexpected Cancers at Final Histology: A Study on Endometrial Sampling Methods and Risk Factors. Diagnostics.

[12] Erdem B., Aşıcıoğlu O., Seyhan N.A., Peker N., Ülker V., Akbayır Ö. (2018). Can concurrent high-risk endometrial carcinoma occur with atypical endometrial hyperplasia?. Int. J. Surg..

[13] https://www.gazzettaufficiale.it/eli/gu/2012/03/26/72/sg/pdf.

[14] Emons G., Beckmann M.W., Schmidt D., Mallmann P. (2015). Uterus commission of the Gynecological Oncology Working Group (AGO). New WHO Classification of Endometrial Hyperplasias. Geburtshilfe Frauenheilkd..

[15] Zaino R.C.S.G., Carinelli S.G., Ellenson L.H., Eng C., Katabuchi H., Konishi I., Lax S., Kurman R.J., Carcanglu M.L., Herrington C.S., Young R.H. (2014). Tumours of the uterine Corpus: Epithelial Tumours and Precursors. WHO Classification of Tumours of female reproductive Organs.

[16] Riley R.D., Ensor J., I E Snell K., E Harrell F., Martin G.P., Reitsma J.B., Moons K.G.M., Collins G., van Smeden M. (2020). Calculating the sample size required for developing a clinical prediction model. BMJ.

[17] Swets J.A. (1988). Measuring the accuracy of diagnostic systems. Science.

[18] Safari S., Baratloo A., Elfil M., Negida A. (2016). Evidence Based Emergency Medicine; Part 5 Receiver Operating Curve and Area under the Curve. Emergency.

[19] Nahm F.S. (2022). Receiver operating characteristic curve: Overview and practical use for clinicians. Korean J. Anesthesiol..

[20] Nees L.K., Heublein S., Steinmacher S., Juhasz-Böss I., Brucker S., Tempfer C.B., Wallwiener M. (2022). Endometrial hyperplasia as a risk factor of endometrial cancer. Arch. Gynecol. Obstet..

[21] Giannella L., Paganelli S. (2017). Abnormal uterine bleeding in premenopausal women and the role of body mass index. Am. J. Obstet. Gynecol..

[22] Wise M.R., Farquhar C.M. (2017). Reply. Am. J. Obstet. Gynecol..

[23] Russo M., Newell J.M., Budurlean L., Houser K.R., Sheldon K., Kesterson J., Phaeton R., Hossler C., Rosenberg J., DeGraff D. (2020). Mutational profile of endometrial hyperplasia and risk of progression to endometrioid adenocarcinoma. Cancer.

[24] Raffone A., Travaglino A., Gabrielli O., Micheli M., Zuccalà V., Bitonti G., Camastra C., Gargiulo V., Insabato L., Zullo F. (2021). Clinical features of ProMisE groups identify different phenotypes of patients with endometrial cancer. Arch. Gynecol. Obstet..

[25] Huang Y.-J., Li B.-L. (2020). The significance of plasma D-dimer level in predicting high risk factors of endometrial cancer. Transl. Cancer Res..

[26] Ge L., Liu G., Hu K., Huang K., Zhang M., Zhou J., Teng F., Cao J., Dai C., Jia X. (2020). A New Risk Index Combining d-Dimer, Fibrinogen, HE4, and CA199 Differentiates Suspecting Endometrial Cancer From Patients With Abnormal Vaginal Bleeding or Discharge. Technol. Cancer Res. Treat..

