# Supplementary material for: Concurrent Endometrial Cancer in Women with Atypical Endometrial Hyperplasia: What Is the Predictive Value of Patient Characteristics?
Source: Cancers (Basel). 2023 Dec 29;16(1):172. doi: 10.3390/cancers16010172 (PMC10778118; doi:10.3390/cancers16010172)
Supplement: Supplementary file 1 [file cancers-16-00172-s001.zip › cancers-2770531-supplementary.pdf]

Table S1. Scheme used to evaluate the performance of prediction models.

	Atypical endometrial hyperplasia	
	Observed not have cancer	Observed have cancer
Predicted not have cancer	TN	FN
Predicted have cancer	FP	TP

This table shows how the results of the predictions of the various algorithms have been divided to evaluate their performance. TN: true negative for cancer, FN: false negative for cancer, FP: false positive for cancer, TP: true positive for cancer. Observed values were based on final histology.

Table S2. Surgical characteristics.

**Independent variables**	**n (%)** **(sample size=629)**
**Surgical approach**	
Laparoscopy	335 (53.3)
Laparotomy	262 (41.7)
Vaginal	32 (5.0)
**Surgical Intervention**	
Hysterectomy	47 (7.5)
Hysterectomy with salpingo-ophorectomy	563 (89.5)
Hysterectomy with salpingectomy	19 (3.0)
**Staging**	
Low-risk	141 (22.4)
Intermediate-risk	26 (4.1)
High-intermediate-risk	19 (3.0)
High-risk	7 (1.2)
No cancer	436 (69.3)

Table S3. Results of the linear regression performed on all number and variable combinations

10-fold cross-validation and linear regression	TRAIN			TEST			More discriminating variables	Explicit variables
Variable number (number of tested combinations)	Mean sensitivity	Mean specificity	Mean AUC	Mean Sensitivity	Mean Specificity	Mean AUC		
2 (120)	60	59.4	0.626 (CI 95%: 0.621-0.631)	59.8	59.1	0.623 (CI 95%: 0.579-0.666)	1, 8	Age, BMI
	50.3	65.8	0.609 (CI 95%: 0.604-0.614)	51.4	66	0.616 (CI 95%: 0.575-0.657)	8, 9	BMI, Lynch
	55.9	60.3	0.617 (CI 95%: 0.612-0.622)	56.3	60.8	0.614 (CI 95%: 0.574-0.653)	8, 15	BMI, RB
	52	61.8	0.601 (CI 95%: 0.597-0.606)	51.2	61.8	0.607 (CI 95%: 0.56-0.654)	5, 8	Bleed, BMI
	55.6	58.8	0.608 (CI 95%: 0.604-0.612)	56	58.1	0.606 (CI 95%: 0.575-0.638)	8, 13	BMI, BC
3 (560)	61.3	61.2	0.645 (CI 95%: 0.638-0.652)	61.9	61.5	0.645 (CI 95%: 0.576-0.714)	1, 8, 9	Age, BMI, Lynch
	61.8	59.7	0.637 (CI 95%: 0.63-0.644)	63.4	59.6	0.637 (CI 95%: 0.562-0.712)	1, 8, 15	Age, BMI, RB
	60.7	59	0.636 (CI 95%: 0.629-0.642)	59.3	58.7	0.631 (CI 95%: 0.568-0.694)	1, 8, 13	Age, BMI, BC
	54.6	64.4	0.631 (CI 95%: 0.623-0.64)	56.6	64.2	0.63 (CI 95%: 0.557-0.704)	8, 9, 15	BMI, Lynch, RB
	59.5	59.7	0.629 (CI 95%: 0.622-0.637)	59.4	59.2	0.63 (CI 95%: 0.555-0.705)	1, 4, 8	Age, Smoke, BMI
4 (1820)	62.6	60.8	0.655 (CI 95%: 0.648-0.662)	60.9	60.8	0.64 (CI 95%: 0.584-0.696)	1, 8, 9, 15	Age, BMI, Lynch, RB
	61.2	60.6	0.653 (CI 95%: 0.647-0.659)	60	60.9	0.639 (CI 95%: 0.584-0.693)	1, 8, 9, 13	Age, BMI, Lynch, BC
	60.6	61.6	0.648 (CI 95%: 0.641-0.654)	59.6	61	0.634 (CI 95%: 0.577-0.691)	1, 8, 9, 10	Age, BMI, Lynch, Tam
	60.4	60.9	0.648 (CI 95%: 0.641-0.655)	58.9	59.7	0.633 (CI 95%: 0.574-0.692)	1, 8, 9, 11	Age, BMI, Lynch, OC
	60.7	61.6	0.648 (CI 95%: 0.642-0.654)	58.6	60.1	0.632 (CI 95%: 0.573-0.692)	1, 7, 8, 9	Age, Dia, BMI, Lynch
5 (4368)	65.6	60.6	0.661 (CI 95%: 0.654-0.669)	64.4	59.3	0.654 (CI 95%: 0.586-0.722)	1, 8, 9, 13, 15	Age, BMI, Lynch, BC, RB
	63.2	61.2	0.656 (CI 95%: 0.65-0.663)	61.7	61.1	0.652 (CI 95%: 0.59-0.714)	1, 5, 8, 9, 13	Age, Bleed, BMI, Lynch, BC
	63.4	62.1	0.66 (CI 95%: 0.653-0.667)	64.1	61.6	0.65 (CI 95%: 0.588-0.713)	1, 5, 8, 9, 15	Age, Bleed, BMI, Lynch, RB
	61	60.3	0.656 (CI 95%: 0.649-0.663)	60.3	59.9	0.649 (CI 95%: 0.584-0.714)	1, 8, 9, 11, 13	Age, BMI, Lynch, OC, BC
	60.8	61.4	0.653 (CI 95%: 0.647-0.659)	59.9	61.8	0.647 (CI 95%: 0.593-0.701)	1, 5, 8, 9, 10	Age, Bleed, BMI, Lynch, Tam
6 (8008)	65.3	60.6	0.663 (CI 95%: 0.656-0.67)	60.5	59.5	0.655 (CI 95%: 0.602-0.709)	1, 7, 8, 9, 13, 15	Age, Dia, BMI, Lynch, BC, RB
	65.6	60.9	0.662 (CI 95%: 0.654-0.669)	61.3	60.6	0.653 (CI 95%: 0.591-0.716)	1, 8, 9, 10, 13, 15	Age, BMI, Lynch, Tam, BC, RB
	63	59.7	0.665 (CI 95%: 0.658-0.671)	60.5	60.1	0.653 (CI 95%: 0.594-0.711)	1, 3, 8, 9, 13, 15	Age, Preg, BMI, Lynch, BC, RB
	66.8	60.3	0.663 (CI 95%: 0.656-0.67)	62	59.7	0.653 (CI 95%: 0.588-0.717)	1, 8, 9, 11, 13, 15	Age, BMI, Lynch, OC, BC, RB
	66.2	60.5	0.661 (CI 95%: 0.654-0.668)	61.7	59.7	0.651 (CI 95%: 0.586-0.715)	1, 2, 8, 9, 13, 15	Age, Mp, BMI, Lynch, BC, RB
7 (11440)	65.3	61.2	0.665 (CI 95%: 0.66-0.67)	62.6	60	0.65 (CI 95%: 0.603-0.697)	1, 5, 7, 8, 9, 11, 15	Age, Bleed, Dia, BMI, Lynch, OC, RB
	65.2	61.5	0.664 (CI 95%: 0.658-0.669)	63.2	60.2	0.648 (CI 95%: 0.602-0.694)	1, 5, 7, 8, 9, 10, 15	Age, Bleed, Dia, BMI, Lynch, Tam, RB
	66	60.9	0.667 (CI 95%: 0.663-0.671)	63.5	60.6	0.648 (CI 95%: 0.612-0.684)	1, 5, 7, 8, 9, 13, 15	Age, Bleed, Dia, BMI, Lynch, BC, RB
	66.7	60.5	0.667 (CI 95%: 0.663-0.671)	63.4	59.9	0.648 (CI 95%: 0.609-0.686)	1, 5, 8, 9, 11, 13, 15	Age, Bleed, BMI, Lynch, OC, BC, RB
	65	61.5	0.663 (CI 95%: 0.657-0.668)	63.5	60.3	0.647 (CI 95%: 0.602-0.692)	1, 5, 8, 9, 10, 11, 15	Age, Bleed, BMI, Lynch, Tam, OC, RB

8 (12870)	63.5	61	0.668 (CI 95%: 0.663-0.674)	60.8	60.4	0.654 (CI 95%: 0.604-0.704)	1, 3, 5, 8, 9, 12, 13, 15	Age, Preg, Bleed, BMI, Lynch, TOS, BC, RB
	63.3	60.6	0.668 (CI 95%: 0.662-0.673)	62	59.4	0.653 (CI 95%: 0.607-0.698)	1, 3, 5, 8, 9, 10, 13, 15	Age, Preg, Bleed, BMI, Lynch, Tam, BC, RB
	64.3	60.7	0.669 (CI 95%: 0.664-0.675)	60.6	59.5	0.652 (CI 95%: 0.603-0.7)	1, 3, 5, 8, 9, 11, 13, 15	Age, Preg, Bleed, BMI, Lynch, OC, BC, RB
	62.6	59.9	0.666 (CI 95%: 0.661-0.672)	61	58.7	0.651 (CI 95%: 0.597-0.705)	1, 3, 8, 9, 11, 12, 13, 15	Age, Preg, BMI, Lynch, OC, TOS, BC, RB
	64.1	61	0.669 (CI 95%: 0.663-0.675)	60.7	60.5	0.651 (CI 95%: 0.603-0.698)	1, 3, 5, 7, 8, 9, 13, 15	Age, Preg, Bleed, Dia, BMI, Lynch, BC, RB
9 (11440)	63.8	60.5	0.669 (CI 95%: 0.664-0.675)	62	60.1	0.654 (CI 95%: 0.606-0.702)	1, 3, 5, 6, 8, 9, 11, 13, 15	Age, Preg, Bleed, Hyp, BMI, Lynch, OC, BC, RB
	64	61.4	0.667 (CI 95%: 0.661-0.672)	61.7	60.9	0.654 (CI 95%: 0.61-0.697)	1, 3, 4, 6, 8, 9, 11, 13, 15	Age, Preg, Smoke, Hyp, BMI, Lynch, OC, BC, RB
	63.8	60.7	0.67 (CI 95%: 0.665-0.675)	63.6	61.2	0.654 (CI 95%: 0.609-0.699)	1, 3, 5, 8, 9, 11, 12, 13, 15	Age, Preg, Bleed, BMI, Lynch, OC, TOS, BC, RB
	64	61.1	0.667 (CI 95%: 0.662-0.672)	60.9	61.6	0.652 (CI 95%: 0.611-0.694)	1, 3, 4, 8, 9, 11, 12, 13, 15	Age, Preg, Smoke, BMI, Lynch, OC, TOS, BC, RB
	64.3	60.3	0.666 (CI 95%: 0.661-0.671)	61.2	60.5	0.652 (CI 95%: 0.609-0.695)	1, 2, 3, 6, 8, 9, 11, 13, 15	Age, Mp, Preg, Hyp, BMI, Lynch, OC, BC, RB
10 (8008)	65.4	60.7	0.671 (CI 95%: 0.663-0.68)	61.8	58.2	0.64 (CI 95%: 0.562-0.718)	1, 3, 4, 5, 8, 9, 10, 11, 13, 15	Age, Preg, Smoke, Bleed, BMI, Lynch, Tam, OC, BC, RB
	66.3	61.1	0.673 (CI 95%: 0.665-0.681)	64.7	58.4	0.64 (CI 95%: 0.563-0.717)	1, 3, 4, 5, 7, 8, 9, 11, 13, 15	Age, Preg, Smoke, Bleed, Dia, BMI, Lynch, OC, BC, RB
	66.2	61.5	0.67 (CI 95%: 0.66-0.679)	62.2	59.9	0.639 (CI 95%: 0.545-0.732)	1, 4, 5, 6, 8, 9, 10, 11, 13, 15	Age, Smoke, Bleed, Hyp, BMI, Lynch, Tam, OC, BC, RB
	66.4	61.7	0.671 (CI 95%: 0.662-0.68)	65.6	61.4	0.638 (CI 95%: 0.546-0.731)	1, 4, 5, 7, 8, 9, 10, 11, 13, 15	Age, Smoke, Bleed, Dia, BMI, Lynch, Tam, OC, BC, RB
	66.9	61.2	0.671 (CI 95%: 0.661-0.68)	65.6	61	0.638 (CI 95%: 0.548-0.729)	1, 2, 4, 5, 7, 8, 9, 11, 13, 15	Age, Mp, Smoke, Bleed, Dia, BMI, Lynch, OC, BC, RB
11 (4368)	63.1	60.9	0.671 (CI 95%: 0.663-0.679)	62.5	59	0.653 (CI 95%: 0.573-0.733)	1, 3, 5, 7, 8, 9, 11, 13, 14, 15, 16	Age, Preg, Bleed, Dia, BMI, Lynch, OC, BC, HB, RB, D&C
	62.1	61.4	0.668 (CI 95%: 0.66-0.677)	61	59.3	0.652 (CI 95%: 0.573-0.73)	1, 3, 4, 5, 7, 8, 9, 11, 14, 15, 16	Age, Preg, Smoke, Bleed, Dia, BMI, Lynch, OC, HB, RB, D&C
	63.1	60.8	0.67 (CI 95%: 0.662-0.678)	62.8	59.9	0.651 (CI 95%: 0.574-0.728)	1, 3, 4, 5, 8, 9, 11, 13, 14, 15, 16	Age, Preg, Smoke, Bleed, BMI, Lynch, OC, BC, HB, RB, D&C
	65.5	60.5	0.67 (CI 95%: 0.662-0.678)	63.3	60.4	0.65 (CI 95%: 0.576-0.724)	1, 4, 5, 7, 8, 9, 11, 13, 14, 15, 16	Age, Smoke, Bleed, Dia, BMI, Lynch, OC, BC, HB, RB, D&C
	64.4	60.4	0.67 (CI 95%: 0.661-0.678)	62.5	58.9	0.65 (CI 95%: 0.571-0.728)	1, 2, 3, 5, 8, 9, 11, 13, 14, 15, 16	Age, Mp, Preg, Bleed, BMI, Lynch, OC, BC, HB, RB, D&C
12 (1820)	65.5	60.6	0.671 (CI 95%: 0.663-0.68)	62	57.5	0.638 (CI 95%: 0.56-0.715)	1, 2, 3, 5, 6, 8, 9, 10, 11, 12, 13, 15	Age, Mp, Preg, Bleed, Hyp, BMI, Lynch, Tam, OC, TOS, BC, RB
	62.8	60.9	0.671 (CI 95%: 0.662-0.68)	57.7	58.8	0.637 (CI 95%: 0.559-0.715)	1, 3, 5, 6, 8, 9, 11, 12, 13, 14, 15, 16	Age, Preg, Bleed, Hyp, BMI, Lynch, OC, TOS, BC, HB, RB, D&C
	62.1	61.2	0.668 (CI 95%: 0.658-0.677)	59.3	59.3	0.636 (CI 95%: 0.549-0.724)	1, 3, 5, 6, 8, 9, 10, 11, 12, 14, 15, 16	Age, Preg, Bleed, Hyp, BMI, Lynch, Tam, OC, TOS, HB, RB, D&C
	63.2	60.6	0.67 (CI 95%: 0.661-0.679)	58.4	58.6	0.636 (CI 95%: 0.559-0.713)	1, 3, 5, 6, 8, 9, 10, 11, 13, 14, 15, 16	Age, Preg, Bleed, Hyp, BMI, Lynch, Tam, OC, BC, HB, RB, D&C
	62	61.8	0.668 (CI 95%: 0.659-0.677)	59.8	59.7	0.636 (CI 95%: 0.55-0.722)	1, 3, 4, 5, 6, 8, 9, 11, 12, 14, 15, 16	Age, Preg, Smoke, Bleed, Hyp, BMI, Lynch, OC, TOS, HB, RB, D&C
13 (560)	65.6	60.9	0.672 (CI 95%: 0.665-0.679)	60.8	57.5	0.632 (CI 95%: 0.572-0.693)	1, 2, 3, 4, 5, 6, 8, 9, 11, 13, 14, 15, 16	Age, Mp, Preg, Smoke, Bleed, Hyp, BMI, Lynch, OC, BC, HB, RB, D&C
	65.9	60.5	0.672 (CI 95%: 0.665-0.678)	60.8	57.3	0.63 (CI 95%: 0.571-0.69)	1, 2, 3, 4, 5, 8, 9, 10, 11, 13, 14, 15, 16	Age, Mp, Preg, Smoke, Bleed, BMI, Lynch, Tam, OC, BC, HB, RB, D&C

	64.9	60.7	0.671 (CI 95%: 0.663-0.679)	60.3	57.4	0.63 (CI 95%: 0.564-0.696)	1, 2, 3, 5, 6, 8, 9, 10, 11, 13, 14, 15, 16	Age, Mp, Preg, Bleed, Hyp, BMI, Lynch, Tam, OC, BC, HB, RB, D&C
	66.8	60.5	0.673 (CI 95%: 0.666-0.681)	61.7	57.9	0.63 (CI 95%: 0.567-0.693)	1, 2, 3, 4, 5, 6, 8, 9, 10, 11, 12, 13, 15	Age, Mp, Preg, Smoke, Bleed, Hyp, BMI, Lynch, Tam, OC, TOS, BC, RB
	63.9	61.1	0.671 (CI 95%: 0.665-0.678)	59.7	58.5	0.63 (CI 95%: 0.57-0.69)	1, 3, 4, 5, 6, 8, 9, 10, 11, 13, 14, 15, 16	Age, Preg, Smoke, Bleed, Hyp, BMI, Lynch, Tam, OC, BC, HB, RB, D&C
14 (120)	64.3	60.9	0.673 (CI 95%: 0.667-0.678)	62.5	55.9	0.63 (CI 95%: 0.581-0.678)	1, 3, 4, 5, 6, 7, 8, 9, 10, 11, 13, 14, 15, 16	Age, Preg, Smoke, Bleed, Hyp, Dia, BMI, Lynch, Tam, OC, BC, HB, RB, D&C
	65.9	61.1	0.674 (CI 95%: 0.668-0.68)	61.3	56.9	0.626 (CI 95%: 0.572-0.68)	1, 2, 3, 4, 5, 6, 7, 8, 9, 11, 13, 14, 15, 16	Age, Mp, Preg, Smoke, Bleed, Hyp, Dia, BMI, Lynch, OC, BC, HB, RB, D&C
	65.8	60.5	0.674 (CI 95%: 0.668-0.68)	62.3	56.7	0.626 (CI 95%: 0.575-0.677)	1, 2, 3, 4, 5, 7, 8, 9, 10, 11, 13, 14, 15, 16	Age, Mp, Preg, Smoke, Bleed, Dia, BMI, Lynch, Tam, OC, BC, HB, RB, D&C
	64.7	61	0.674 (CI 95%: 0.668-0.68)	61.3	56.1	0.626 (CI 95%: 0.567-0.685)	1, 3, 4, 5, 6, 7, 8, 9, 11, 12, 13, 14, 15, 16	Age, Preg, Smoke, Bleed, Hyp, Dia, BMI, Lynch, OC, TOS, BC, HB, RB, D&C
	65.5	61.1	0.672 (CI 95%: 0.667-0.678)	60.8	57.9	0.626 (CI 95%: 0.576-0.675)	1, 2, 3, 4, 5, 6, 8, 9, 10, 11, 13, 14, 15, 16	Age, Mp, Preg, Smoke, Bleed, Hyp, BMI, Lynch, Tam, OC, BC, HB, RB, D&C
15 (16)	64.8	61	0.674 (CI 95%: 0.666-0.682)	60.8	58.1	0.626 (CI 95%: 0.555-0.696)	1, 3, 4, 5, 6, 7, 8, 9, 10, 11, 12, 13, 14, 15, 16	Age, Preg, Smoke, Bleed, Hyp, Dia, BMI, Lynch, Tam, OC, TOS, BC, HB, RB, D&C
	66.1	60.7	0.675 (CI 95%: 0.667-0.683)	61.5	57	0.623 (CI 95%: 0.55-0.697)	1, 2, 3, 4, 5, 6, 7, 8, 9, 11, 12, 13, 14, 15, 16	Age, Mp, Preg, Smoke, Bleed, Hyp, Dia, BMI, Lynch, OC, TOS, BC, HB, RB, D&C
	66.1	60.7	0.674 (CI 95%: 0.666-0.682)	62.7	57.2	0.623 (CI 95%: 0.553-0.693)	1, 2, 3, 4, 5, 6, 7, 8, 9, 10, 11, 13, 14, 15, 16	Age, Mp, Preg, Smoke, Bleed, Hyp, Dia, BMI, Lynch, Tam, OC, BC, HB, RB, D&C
	64.3	60.7	0.673 (CI 95%: 0.664-0.682)	61.2	57.9	0.623 (CI 95%: 0.543-0.703)	1, 2, 3, 4, 5, 6, 7, 8, 9, 10, 11, 12, 14, 15, 16	Age, Mp, Preg, Smoke, Bleed, Hyp, Dia, BMI, Lynch, Tam, OC, TOS, HB, RB, D&C
	66.1	60.9	0.675 (CI 95%: 0.667-0.683)	63.2	56.7	0.623 (CI 95%: 0.548-0.697)	1, 2, 3, 4, 5, 7, 8, 9, 10, 11, 12, 13, 14, 15, 16	Age, Mp, Preg, Smoke, Bleed, Dia, BMI, Lynch, Tam, OC, TOS, BC, HB, RB, D&C

1 Age; 2 Menopause (Mp); 3 previous pregnancies (Preg); 4 Smoking habit (Smoke); 5 abnormal uterine bleeding (bleed); 6 hypertension (Hyp); 7 diabet (Dia); 8 BMI; 9 hereditary Lynch syndrome (Lynch); 10 Previous Tamoxifen therapy (Tam); 11 hormonal therapy with OC (OC); 12 hormonal therapy use TOS (TOS); 13 previous breast cancer (BC); 14 hysteroscopically guided biopsy (HB); 15 hysteroscopic endometrial resection (RB); 16 D&C dilation and curettage.

Table S4. Results from Support Vector Machine-based predictors.

10-fold cross-validation and SVM	TRAIN			TEST			More discriminating variables	Explicit variables
Variable number (number of tested combinations)	Mean sensitivity	Mean specificity	Mean AUC	Mean Sensitivity	Mean Specificity	Mean AUC		
3 (560)	25.3	96.3	0.621 (CI 95%: 0.609-0.632)	20.9	92	0.59 (CI 95%: 0.555-0.624)	3, 8, 9	Preg, BMI, Lynch
	23.8	96.3	0.617 (CI 95%: 0.6-0.633)	19.3	90.1	0.584 (CI 95%: 0.532-0.637)	3, 8, 12	Preg, BMI, TOS
	31.3	94.4	0.642 (CI 95%: 0.632-0.653)	25.3	87.3	0.584 (CI 95%: 0.531-0.636)	3, 8, 16	Preg, BMI, D&C
	86.8	97.4	0.967 (CI 95%: 0.962-0.971)	51.6	56.9	0.582 (CI 95%: 0.548-0.616)	1, 3, 8	Age, Preg, BMI
	29.1	93.7	0.622 (CI 95%: 0.603-0.64)	21.6	89.6	0.573 (CI 95%: 0.516-0.63)	4, 8, 9	Smoke, BMI, Lynch
4 (1820)	32.2	94.8	0.651 (CI 95%: 0.638-0.664)	21.9	85.3	0.597 (CI 95%: 0.535-0.658)	3, 8, 10, 16	Preg, BMI, Tam, D&C
	44.1	89.2	0.673 (CI 95%: 0.652-0.694)	29.7	80.2	0.59 (CI 95%: 0.539-0.641)	1, 9, 12, 15	Age, Lynch, TOS, RB
	46.6	88.4	0.689 (CI 95%: 0.678-0.7)	29.2	79.1	0.584 (CI 95%: 0.532-0.635)	1, 2, 9, 15	Age, Mp, Lynch, RB
	31.7	93.7	0.631 (CI 95%: 0.622-0.641)	24.6	87	0.582 (CI 95%: 0.509-0.655)	4, 8, 9, 12	Smoke, BMI, Lynch, TOS
	30.4	93.9	0.632 (CI 95%: 0.617-0.647)	21.9	86.8	0.582 (CI 95%: 0.521-0.644)	4, 8, 10, 12	Smoke, BMI, Tam, TOS
5 (4368)	53.8	89.9	0.737 (CI 95%: 0.727-0.747)	38.6	76.4	0.622 (CI 95%: 0.574-0.67)	1, 2, 9, 13, 14	Age, Mp, Lynch, BC, HB
	44.6	92.8	0.707 (CI 95%: 0.69-0.724)	35.7	78.6	0.607 (CI 95%: 0.566-0.649)	1, 7, 9, 13, 16	Age, Dia, Lynch, BC, D&C
	48.9	91.6	0.732 (CI 95%: 0.726-0.738)	37	78.6	0.606 (CI 95%: 0.558-0.654)	2, 4, 5, 8, 10	Mp, Smoke, Bleed, BMI, Tam
	54.4	89.5	0.733 (CI 95%: 0.723-0.743)	41.4	75.2	0.605 (CI 95%: 0.561-0.649)	1, 9, 13, 14, 15	Age, Lynch, BC, HB, RB
	49.3	88.5	0.707 (CI 95%: 0.694-0.721)	35.7	78.6	0.605 (CI 95%: 0.556-0.653)	1, 2, 9, 12, 15	Age, Mp, Lynch, TOS, RB
6 (8008)	51	92.2	0.741 (CI 95%: 0.736-0.747)	39.1	78.2	0.611 (CI 95%: 0.547-0.675)	2, 4, 5, 8, 9, 10	Mp, Smoke, Bleed, BMI, Lynch, Tam
	48.8	93.7	0.725 (CI 95%: 0.715-0.735)	33.9	77.2	0.611 (CI 95%: 0.549-0.672)	3, 4, 7, 8, 9, 16	Preg, Smoke, Dia, BMI, Lynch, D&C
	54.2	93.8	0.77 (CI 95%: 0.76-0.78)	39.4	74.2	0.611 (CI 95%: 0.556-0.665)	3, 5, 6, 8, 9, 16	Preg, Bleed, Hyp, BMI, Lynch, D&C
	60.7	94.3	0.817 (CI 95%: 0.809-0.824)	45.8	70.4	0.607 (CI 95%: 0.559-0.656)	3, 4, 5, 6, 8, 16	Preg, Smoke, Bleed, Hyp, BMI, D&C
	54.9	93.2	0.79 (CI 95%: 0.779-0.802)	38.6	73.7	0.605 (CI 95%: 0.558-0.651)	4, 5, 6, 8, 11, 16	Smoke, Bleed, Hyp, BMI, OC, D&C
7 (11440)	51.3	92.7	0.759 (CI 95%: 0.749-0.77)	39.9	74.3	0.613 (CI 95%: 0.563-0.664)	2, 5, 8, 10, 12, 13, 16	Mp, Bleed, BMI, Tam, TOS, BC, D&C
	62.3	94.5	0.836 (CI 95%: 0.828-0.843)	49.7	70.1	0.613 (CI 95%: 0.568-0.658)	3, 4, 5, 6, 8, 9, 16	Preg, Smoke, Bleed, Hyp, BMI, Lynch, D&C
	95.5	98.7	0.992 (CI 95%: 0.99-0.994)	57.9	56.4	0.612 (CI 95%: 0.567-0.657)	1, 3, 4, 8, 9, 11, 15	Age, Preg, Smoke, BMI, Lynch, OC, RB
	96.1	98.6	0.994 (CI 95%: 0.991-0.996)	61	54.8	0.61 (CI 95%: 0.567-0.652)	1, 3, 4, 7, 8, 9, 15	Age, Preg, Smoke, Dia, BMI, Lynch, RB
	58.7	93.4	0.8 (CI 95%: 0.794-0.806)	48.2	72.3	0.609 (CI 95%: 0.559-0.659)	3, 4, 6, 7, 8, 9, 16	Preg, Smoke, Hyp, Dia, BMI, Lynch, D&C
8 (12870)	95.8	98.7	0.995 (CI 95%: 0.993-0.997)	61.9	56.5	0.608 (CI 95%: 0.551-0.664)	1, 3, 4, 7, 8, 9, 11, 15	Age, Preg, Smoke, Dia, BMI, Lynch, OC, RB
	60.6	93.9	0.81 (CI 95%: 0.799-0.82)	44.2	71	0.601 (CI 95%: 0.539-0.663)	3, 4, 6, 7, 8, 9, 12, 16	Preg, Smoke, Hyp, Dia, BMI, Lynch, TOS, D&C
	66.4	92.4	0.834 (CI 95%: 0.825-0.843)	47.8	67.8	0.601 (CI 95%: 0.55-0.652)	3, 4, 5, 7, 8, 9, 11, 14	Preg, Smoke, Bleed, Dia, BMI, Lynch, OC, HB
	63.7	94.9	0.841 (CI 95%: 0.829-0.853)	47	67.1	0.601 (CI 95%: 0.558-0.643)	3, 4, 5, 6, 8, 9, 12, 16	Preg, Smoke, Bleed, Hyp, BMI, Lynch, TOS, D&C

	96.4	98.6	0.995 (CI 95%: 0.993-0.997)	61.1	55.1	0.6 (CI 95%: 0.538-0.661)	1, 3, 4, 7, 8, 9, 12, 15	Age, Preg, Smoke, Dia, BMI, Lynch, TOS, RB
9 (11440)	73.6	92.6	0.874 (CI 95%: 0.865-0.882)	47.6	65	0.605 (CI 95%: 0.55-0.66)	2, 4, 5, 7, 8, 9, 10, 13, 14	Mp, Smoke, Bleed, Dia, BMI, Lynch, Tam, BC, HB
	77.4	93.2	0.912 (CI 95%: 0.904-0.921)	52.7	62.9	0.596 (CI 95%: 0.556-0.636)	2, 3, 4, 5, 7, 8, 9, 13, 14	Mp, Preg, Smoke, Bleed, Dia, BMI, Lynch, BC, HB
	97.8	98.2	0.992 (CI 95%: 0.99-0.994)	54.8	58.3	0.595 (CI 95%: 0.543-0.647)	1, 3, 7, 8, 11, 12, 13, 15, 16	Age, Preg, Dia, BMI, OC, TOS, BC, RB, D&C
	97.4	98.4	0.995 (CI 95%: 0.993-0.996)	58.3	56.1	0.594 (CI 95%: 0.534-0.653)	1, 3, 7, 8, 9, 12, 13, 15, 16	Age, Preg, Dia, BMI, Lynch, TOS, BC, RB, D&C
	59.2	94.1	0.822 (CI 95%: 0.808-0.835)	43.9	67.2	0.592 (CI 95%: 0.51-0.674)	4, 5, 6, 8, 9, 10, 11, 12, 16	Smoke, Bleed, Hyp, BMI, Lynch, Tam, OC, TOS, D&C
10 (8008)	63.3	95.3	0.835 (CI 95%: 0.818-0.852)	49.7	60.8	0.605 (CI 95%: 0.55-0.661)	3, 4, 6, 7, 8, 9, 11, 12, 13, 16	Preg, Smoke, Hyp, Dia, BMI, Lynch, OC, TOS, BC, D&C
	63.7	95.1	0.84 (CI 95%: 0.834-0.846)	50.1	64.3	0.601 (CI 95%: 0.547-0.656)	3, 4, 6, 7, 8, 9, 10, 12, 13, 16	Preg, Smoke, Hyp, Dia, BMI, Lynch, Tam, TOS, BC, D&C
	70.7	93.3	0.858 (CI 95%: 0.845-0.872)	48.8	65.4	0.588 (CI 95%: 0.54-0.636)	3, 4, 5, 7, 8, 9, 10, 11, 13, 14	Preg, Smoke, Bleed, Dia, BMI, Lynch, Tam, OC, BC, HB
	76.9	94.4	0.901 (CI 95%: 0.894-0.907)	56.3	61.7	0.586 (CI 95%: 0.521-0.652)	2, 3, 4, 5, 6, 7, 8, 9, 12, 16	Mp, Preg, Smoke, Bleed, Hyp, Dia, BMI, Lynch, TOS, D&C
	80	93.1	0.915 (CI 95%: 0.91-0.921)	53.3	62.9	0.585 (CI 95%: 0.543-0.626)	2, 3, 4, 5, 7, 8, 9, 11, 13, 14	Mp, Preg, Smoke, Bleed, Dia, BMI, Lynch, OC, BC, HB
11 (4368)	98	99.2	1 (CI 95%: 1-1)	56.4	62.3	0.609 (CI 95%: 0.549-0.668)	1, 2, 4, 5, 7, 8, 11, 13, 14, 15, 16	Age, Mp, Smoke, Bleed, Dia, BMI, OC, BC, HB, RB, D&C
	97.9	99.2	1 (CI 95%: 1-1)	57.7	61.8	0.608 (CI 95%: 0.546-0.67)	1, 2, 4, 5, 7, 8, 9, 11, 13, 14, 16	Age, Mp, Smoke, Bleed, Dia, BMI, Lynch, OC, BC, HB, D&C
	98	99.2	1 (CI 95%: 1-1)	54.7	62.8	0.607 (CI 95%: 0.55-0.664)	1, 2, 4, 5, 7, 8, 10, 11, 13, 14, 16	Age, Mp, Smoke, Bleed, Dia, BMI, Tam, OC, BC, HB, D&C
	98.4	99.6	1 (CI 95%: 1-1)	51.3	64.1	0.606 (CI 95%: 0.565-0.648)	1, 2, 3, 4, 5, 7, 8, 11, 13, 14, 15	Age, Mp, Preg, Smoke, Bleed, Dia, BMI, OC, BC, HB, RB
	98	99.2	1 (CI 95%: 1-1)	51.1	62.7	0.605 (CI 95%: 0.552-0.658)	1, 2, 4, 5, 7, 8, 10, 11, 13, 14, 15	Age, Mp, Smoke, Bleed, Dia, BMI, Tam, OC, BC, HB, RB
12 (1820)	98.4	99.7	1 (CI 95%: 1-1)	50.5	62.7	0.597 (CI 95%: 0.552-0.643)	1, 2, 3, 4, 5, 7, 8, 11, 13, 14, 15, 16	Age, Mp, Preg, Smoke, Bleed, Dia, BMI, OC, BC, HB, RB, D&C
	96.8	99.3	0.996 (CI 95%: 0.995-0.998)	65.5	52.4	0.592 (CI 95%: 0.532-0.651)	1, 2, 3, 5, 7, 8, 9, 10, 11, 12, 13, 14	Age, Mp, Preg, Bleed, Dia, BMI, Lynch, Tam, OC, TOS, BC, HB
	97.9	99.5	1 (CI 95%: 1-1)	58.6	59.1	0.589 (CI 95%: 0.53-0.648)	1, 2, 3, 4, 5, 7, 8, 9, 11, 12, 13, 14	Age, Mp, Preg, Smoke, Bleed, Dia, BMI, Lynch, OC, TOS, BC, HB
	98.5	99.6	1 (CI 95%: 1-1)	47.1	64.9	0.589 (CI 95%: 0.545-0.632)	1, 2, 3, 4, 5, 7, 8, 11, 12, 13, 14, 15	Age, Mp, Preg, Smoke, Bleed, Dia, BMI, OC, TOS, BC, HB, RB
	80.8	93.5	0.92 (CI 95%: 0.915-0.924)	56.6	61.9	0.587 (CI 95%: 0.546-0.629)	2, 3, 4, 5, 7, 8, 9, 10, 11, 12, 13, 14	Mp, Preg, Smoke, Bleed, Dia, BMI, Lynch, Tam, OC, TOS, BC, HB
13 (560)	81	94.7	0.931 (CI 95%: 0.923-0.938)	51.6	60.7	0.58 (CI 95%: 0.52-0.64)	2, 3, 4, 5, 7, 8, 9, 10, 11, 12, 13, 15, 16	Mp, Preg, Smoke, Bleed, Dia, BMI, Lynch, Tam, OC, TOS, BC, RB, D&C
	98	99.2	1 (CI 95%: 1-1)	50.8	59.4	0.577 (CI 95%: 0.517-0.638)	1, 2, 4, 5, 7, 8, 9, 10, 11, 13, 14, 15, 16	Age, Mp, Smoke, Bleed, Dia, BMI, Lynch, Tam, OC, BC, HB, RB, D&C
	98	99.2	1 (CI 95%: 1-1)	49.6	61.7	0.576 (CI 95%: 0.519-0.633)	1, 2, 4, 5, 7, 8, 9, 10, 11, 12, 13, 14, 15	Age, Mp, Smoke, Bleed, Dia, BMI, Lynch, Tam, OC, TOS, BC, HB, RB
	98	99.2	1 (CI 95%: 1-1)	50.4	59.2	0.576 (CI 95%: 0.515-0.637)	1, 2, 4, 5, 7, 8, 9, 11, 12, 13, 14, 15, 16	Age, Mp, Smoke, Bleed, Dia, BMI, Lynch, OC, TOS, BC, HB, RB, D&C

	80.7	94.6	0.922 (CI 95%: 0.915-0.93)	50.8	61.8	0.576 (CI 95%: 0.509-0.642)	2, 3, 4, 5, 7, 8, 9, 11, 12, 13, 14, 15, 16	Mp, Preg, Smoke, Bleed, Dia, BMI, Lynch, OC, TOS, BC, HB, RB, D&C
14 (120)	98	99.2	1 (CI 95%: 1-1)	49.3	60.8	0.585 (CI 95%: 0.514-0.657)	1, 2, 4, 5, 7, 8, 9, 10, 11, 12, 13, 14, 15, 16	Age, Mp, Smoke, Bleed, Dia, BMI, Lynch, Tam, OC, TOS, BC, HB, RB, D&C
	99.1	100	1 (CI 95%: 1-1)	50.8	61.1	0.577 (CI 95%: 0.52-0.635)	1, 2, 3, 4, 5, 6, 7, 8, 9, 10, 11, 13, 15, 16	Age, Mp, Preg, Smoke, Bleed, Hyp, Dia, BMI, Lynch, Tam, OC, BC, RB, D&C
	98.6	99.6	1 (CI 95%: 1-1)	50.6	60.3	0.577 (CI 95%: 0.521-0.632)	1, 2, 3, 4, 5, 7, 8, 9, 10, 11, 12, 13, 15, 16	Age, Mp, Preg, Smoke, Bleed, Dia, BMI, Lynch, Tam, OC, TOS, BC, RB, D&C
	98.3	99.7	1 (CI 95%: 1-1)	49.3	61	0.576 (CI 95%: 0.521-0.632)	1, 2, 3, 4, 5, 7, 8, 9, 10, 11, 12, 13, 14, 16	Age, Mp, Preg, Smoke, Bleed, Dia, BMI, Lynch, Tam, OC, TOS, BC, HB, D&C
	99.1	100	1 (CI 95%: 1-1)	50.4	62.3	0.576 (CI 95%: 0.516-0.636)	1, 2, 3, 4, 5, 6, 7, 8, 9, 11, 12, 13, 15, 16	Age, Mp, Preg, Smoke, Bleed, Hyp, Dia, BMI, Lynch, OC, TOS, BC, RB, D&C
15 (16)	99.1	100	1 (CI 95%: 1-1)	50	62.8	0.586 (CI 95%: 0.525-0.647)	1, 2, 3, 4, 5, 6, 7, 8, 9, 11, 12, 13, 14, 15, 16	Age, Mp, Preg, Smoke, Bleed, Hyp, Dia, BMI, Lynch, OC, TOS, BC, HB, RB, D&C
	98.3	99.7	1 (CI 95%: 1-1)	51.7	61.6	0.585 (CI 95%: 0.543-0.627)	1, 2, 3, 4, 5, 7, 8, 9, 10, 11, 12, 13, 14, 15, 16	Age, Mp, Preg, Smoke, Bleed, Dia, BMI, Lynch, Tam, OC, TOS, BC, HB, RB, D&C
	99.1	100	1 (CI 95%: 1-1)	49.3	63.5	0.585 (CI 95%: 0.517-0.653)	1, 2, 3, 4, 5, 6, 7, 8, 9, 10, 11, 12, 13, 14, 16	Age, Mp, Preg, Smoke, Bleed, Hyp, Dia, BMI, Lynch, Tam, OC, TOS, BC, HB, D&C
	99.1	100	1 (CI 95%: 1-1)	51.4	63.7	0.585 (CI 95%: 0.521-0.65)	1, 2, 3, 4, 5, 6, 7, 8, 9, 10, 11, 12, 13, 15, 16	Age, Mp, Preg, Smoke, Bleed, Hyp, Dia, BMI, Lynch, Tam, OC, TOS, BC, RB, D&C
	99.1	100	1 (CI 95%: 1-1)	48.1	65.1	0.584 (CI 95%: 0.536-0.632)	1, 2, 3, 4, 5, 6, 8, 9, 10, 11, 12, 13, 14, 15, 16	Age, Mp, Preg, Smoke, Bleed, Hyp, BMI, Lynch, Tam, OC, TOS, BC, HB, RB, D&C

1 Age; 2 Menopause (Mp); 3 previous pregnancies (Preg); 4 Smoking habit (Smoke); 5 abnormal uterine bleeding (bleed); 6 hypertension (Hyp); 7 diabet (Dia); 8 BMI; 9 hereditary Lynch syndrome (Lynch); 10 Previous Tamoxifen therapy (Tam); 11 hormonal therapy with OC (OC); 12 hormonal therapy use TOS (TOS); 13 previous breast cancer (BC); 14 hysteroscopically guided biopsy (HB); 15 hysteroscopic endometrial resection (RB); 16 D&C dilation and curettage. SVM algorithm results. It is noted that the performance on the training set is much better than on the test set, so overfitting has occurred. Several settings were used, but the overfitting remained.

Figure S1. Comparative analysis of three different functions.

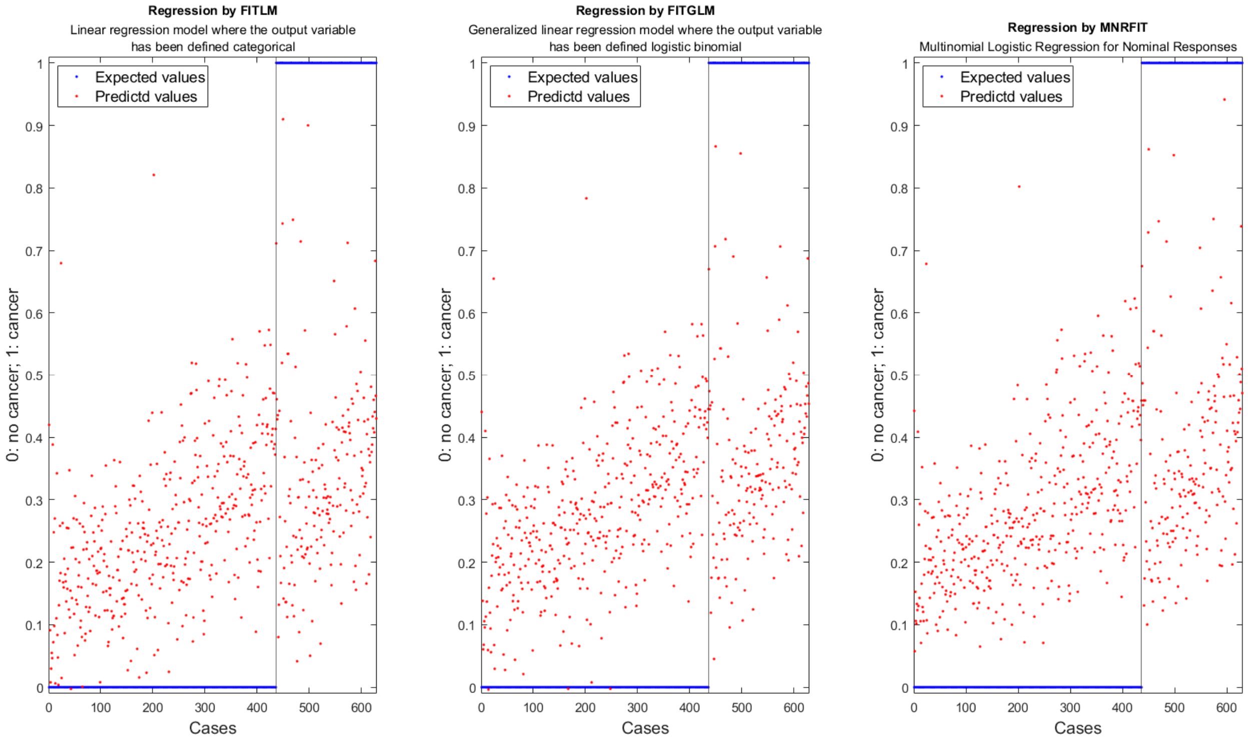

Comparative analysis of three different functions available in Matlab to implement logistic regressions. The 629 patients are shown on the abscissa axis. The output variable, i.e., the presence of cancer, is reported in the ordinate axis. The data are sorted by output variable whereby the blue horizontal lines represent 436 patients without cancer and 193 patients with cancer. All variables were used to perform the regressions. It is noted that the results are highly similar.
